# Asymptotic expansions for partitions generated by infinite products

**DOI:** 10.1007/s00208-024-02807-x

**Published:** 2024-02-26

**Authors:** Walter Bridges, Benjamin Brindle, Kathrin Bringmann, Johann Franke

**Affiliations:** Department of Mathematics and Computer Science, University of Cologne, Weyertal 86-90, 50931 Cologne, Germany

**Keywords:** 11E45, 11M41, 11P82

## Abstract

Recently, Debruyne and Tenenbaum proved asymptotic formulas for the number of partitions with parts in $\Lambda \subset {\mathbb {N}}$ ($\gcd (\Lambda )=1$) and good analytic properties of the corresponding zeta function, generalizing work of Meinardus. In this paper, we extend their work to prove asymptotic formulas if $\Lambda$ is a multiset of integers and the zeta function has multiple poles. In particular, our results imply an asymptotic formula for the number of irreducible representations of degree *n* of ${\mathfrak {so}{(5)}}$. We also study the Witten zeta function $\zeta _{{\mathfrak {so}{(5)}}}$, which is of independent interest.

## Introduction and statement of results

### The circle method

In analytic number theory and combinatorics, one uses complex analysis to better understand properties of sequences. Suppose that a sequence $(c(n))_{n\in {\mathbb {N}}_0}$ has moderate growth and the *generating function*

$$\begin{aligned} F(q) :=\sum _{n \ge 0} c(n)q^n, \end{aligned}$$

is holomorphic in the unit disk with radius of convergence 1. Via Cauchy’s integral formula one can then recover the coefficients from the generating function

1.1 $$\begin{aligned} c(n) = \frac{1}{2\pi i} \int _{{\mathcal {C}}} \frac{F(q)}{q^{n+1}} dq, \end{aligned}$$

for any simple closed curve ${\mathcal {C}}$ contained in the unit disk orientated counterclockwise. The so-called circle method uses the analytic behavior of *F*(*q*) near the boundary of the unit circle to obtain asymptotic information about *c*(*n*). In fact for “nice” examples this method is automatic and there is a long history for example with the prime number theorem. For instance, if the *c*(*n*) are positive and monotonically increasing, it is expected that the part close to $q=1$ provides the dominant contribution to (1.1) (Tauberian Theorems then show this). This part of the curve is the *major arc* and the complement is the *minor arc*. To obtain an asymptotic expansion for *c*(*n*), one then evaluates the major arc to some degree of accuracy and bounds the minor arc. Depending on the function *F*(*q*), both of these tasks present a variety of difficulties.

In the present paper, we are interested in infinite product generating functions of the form

$$\begin{aligned} F(q) = \prod _{n \ge 1} \frac{1}{(1 - q^n)^{f(n)}}. \end{aligned}$$

Such generating functions are important in the theory of partitions, but also arise, for example, in representation theory. If the Dirichlet series for *f*(*n*) has a single simple pole on the positive real axis and *F* is “bounded” away from $q=1$, then Meinardus [30] proved an asymptotic expression for *c*(*n*). Debruyne and Tenenbaum [17] eliminated the technical growth conditions on *F* by adding a few more assumptions on the *f*(*n*), which made their result more applicable. Our main results, Theorems 1.4 and 4.4, yield asymptotic expansions given mild assumptions on *f*(*n*) and have a variety of new applications.

### The classical partition function

Let $n \in {\mathbb {N}}$. A weakly decreasing sequence of positive integers that sum to *n* is called a *partition* of *n*. The number of partitions is denoted by *p*(*n*). If $\lambda _1+\cdots +\lambda _r=n$, then the $\lambda _j$ are called the *parts* of the partition. The partition function has no elementary closed formula, nor does it satisfy any finite order recurrence. However, setting $p(0):=1$, its generating function has the following product expansion

1.2 $$\begin{aligned} \sum _{n\ge 0} p(n)q^n = \prod _{n\ge 1} \frac{1}{1-q^n}, \end{aligned}$$

where $|q| < 1$. In [23], Hardy and Ramanujan used (1.2) to show the asymptotic formula

$$\begin{aligned} p(n) \sim \frac{1}{4\sqrt{3}n}e^{\pi \sqrt{\frac{2n}{3}}},\qquad n\rightarrow \infty , \end{aligned}$$

which gave birth of the Circle Method. Using modular transformations one can describe with high precision the analytic behavior of the product if *q* is near a root of unity. One further sees directly from the infinite product that dominant singularities occur at such roots of unity with small denominator. These ideas culminate in Rademacher’s exact formula for *p*(*n*) [33].

With Theorem 1.4 we find, for certain constants $B_j$ and arbitrary $N\in {\mathbb {N}}$,

1.3 $$\begin{aligned} p(n) = \frac{e^{\pi \sqrt{\frac{2n}{3}}}}{4\sqrt{3}n}\left( 1+\sum _{j=1}^N \frac{B_j}{n^\frac{j}{2}}+O_N\left( n^{-\frac{N+1}{2}}\right) \right) . \end{aligned}$$

Similarly, one can treat the cases for *k*-th powers (in arithmetic progressions), see [17].

### Plane partitions

Another application is an asymptotic formula for plane partitions. A *plane partition of size n* is a two-dimensional array of non-negative integers $\pi _{j,k}$ for which $\sum _{j,k}\pi _{j,k}=n$, such that $\pi _{j,k}\ge \pi _{j,k+1}$ and $\pi _{j,k}\ge \pi _{j+1,k}$ for all $j,k\in {\mathbb {N}}$. We denote the number of plane partitions of *n* by ${\text {pp}}(n)$. MacMahon [25] proved that

$$\begin{aligned} \sum _{n\ge 0} {\text {pp}}(n)q^n = \prod _{n\ge 1} \frac{1}{\left( 1-q^n\right) ^n}. \end{aligned}$$

Using Theorem 1.4, we recover Wright’s asymptotic formula^1^ [38]

$$\begin{aligned} {\text {pp}}(n) = \frac{C}{n^\frac{25}{36}} e^{A_1 n^\frac{2}{3}}\left( 1+\sum _{j=2}^{N+1} \frac{B_j}{n^\frac{2(j-1)}{3}}+O_N\left( n^{-\frac{2(N+1)}{3}}\right) \right) , \end{aligned}$$

where the constants $B_j$ are explicitly computable,

$$\begin{aligned} C:=\frac{\zeta (3)^\frac{7}{36}e^{\zeta '(-1)}}{2^{\frac{11}{36}}\sqrt{3\pi }}, \qquad A_1:=\frac{3\zeta (3)^\frac{1}{3}}{2^\frac{2}{3}} \end{aligned}$$

with $\zeta$ the Riemann zeta function.

### Partitions into polygonal numbers

The *n*-th *k* *-gonal number* is given by^2^ ($k\in {\mathbb {N}}_{\ge 3}$)

1.4 $$\begin{aligned} P_k(n) := \frac{1}{2} \left( (k-2)n^2 +(4-k)n\right) . \end{aligned}$$

The study of representations of integers as sums of polygonal numbers has a long history. Fermat conjectured in 1638 that every $n\in {\mathbb {N}}$ may be written as the sum of at most *k* *k*-gonal numbers which was finally proved by Cauchy. Let $p_k(n)$ denotes the number of partitions of *n* into *k*-gonal numbers. We have the generating function

$$\begin{aligned} \sum _{n \ge 0} p_k(n)q^n = \prod _{n \ge 1} \frac{1}{1-q^{P_k(n)}}. \end{aligned}$$

The $p_k(n)$ have the following asymptotics.^3^

#### Theorem 1.1

We have, for all^4^$N\in {\mathbb {N}}$,

$$\begin{aligned} p_k(n) = \frac{C(k)e^{A(k)n^\frac{1}{3}}}{n^\frac{5k-6}{6(k-2)}}\left( 1+\sum _{j=1}^N \frac{B_{j,k}}{n^\frac{j}{3}}+O_N\left( n^{-\frac{N+1}{3}}\right) \right) , \end{aligned}$$

where the $B_{j,k}$ can be computed explicitly and

$$\begin{aligned} C(k) := \frac{(k-2)^\frac{6-k}{6(k-2)}\Gamma \left( \frac{2}{k-2}\right) \zeta \left( \frac{3}{2}\right) ^\frac{k}{3(k-2)}}{2^{\frac{3k-2}{2(k-2)}}\sqrt{3}\pi ^\frac{4k-9}{3(k-2)}},\qquad A(k) := \frac{3}{2}\left( \sqrt{\frac{\pi }{k-2}}\zeta \left( \frac{3}{2}\right) \right) ^\frac{2}{3}. \end{aligned}$$

#### Remark

Theorem 1.1 strengthens an asymptotic formula of Brigham for $\log (p_k(n))$ (see page 191 of [7] part D).

### Numbers of finite-dimensional representations of Lie algebras

The special unitary group $\mathfrak {su}(2)$ has (up to equivalence) one irreducible representation $V_k$ of each dimension $k\in {\mathbb {N}}$. Each *n*-dimensional representation $\bigoplus _{k=1}^\infty r_k V_k$ corresponds to a unique partition

1.5 $$\begin{aligned} n = \lambda _1 + \lambda _2 + \cdots + \lambda _r, \qquad \lambda _1 \ge \lambda _2 \ge \cdots \ge \lambda _r \ge 1 \end{aligned}$$

such that $r_k$ counts the number of *k* in (1.5). As a result, the number of representations equals *p*(*n*). It is natural to ask whether this can be generalized. The next case is the unitary group $\mathfrak {su}(3)$, whose irreducible representations $W_{j,k}$ indexed by pairs of positive integers. Note that (see Chapter 5 of [22]) $\dim (W_{j,k})=\frac{1}{2}jk(j+k)$. Like in the case of $\mathfrak {su}(2)$, a general *n*-dimensional representation decomposes into a sum of these $W_{j,k}$, again each with some multiplicity. So analogous to (1.2), the numbers $r_{\mathfrak {su}(3)}(n)$ of *n*-dimensional representations, have the generating function

$$\begin{aligned} \sum _{n\ge 0} r_{\mathfrak {su}(3)}(n)q^n = \prod _{j,k\ge 1} \frac{1}{1-q^{\frac{jk(j+k)}{2}}}, \end{aligned}$$

again with $r_{\mathfrak {su}(3)}(0):=1$. In [34], Romik proved that, as $n\rightarrow \infty$,

$$\begin{aligned} r_{\mathfrak {su}(3)}(n) \sim \frac{C_0}{n^{\frac{3}{5}}} \exp \left( A_1n^{\frac{2}{5}}+A_2n^{\frac{3}{10}}+A_3n^{\frac{1}{5}}+A_4n^{\frac{1}{10}}\right) , \end{aligned}$$

with explicit constants^5^$C_0,A_1,\dots ,A_4$ expressible in terms of zeta and gamma values. Two of the authors [8] improved this to an analogue of formula (1.3), namely, for any $N\in {\mathbb {N}}_0$, we have

1.6 $$\begin{aligned} r_{\mathfrak {su}(3)}(n)&= \frac{C_0}{n^{\frac{3}{5}}}\exp \left( A_1n^{\frac{2}{5}}+A_2n^{\frac{3}{10}}+A_3n^{\frac{1}{5}}+A_4n^{\frac{1}{10}}\right) \nonumber \\&\quad \times \left( 1+\sum _{j=1}^{N}\frac{C_j}{n^\frac{j}{10}} + O_N\left( n^{-\frac{N+1}{10}}\right) \right) , \end{aligned}$$

as $n\rightarrow \infty$, where the constants $C_j$ do not depend on *N* and *n* and can be calculated explicitly. The expansion (1.6) with explicit values for $A_j$ ($1\le j\le 4$) and $C_0$, can also be obtained using Theorem 4.4.

This framework generalizes to other groups. For example, one can investigate the *Witten zeta function* for $\mathfrak {so}(5)$, which is (for more background to this function, see [27, 28])

1.7 $$\begin{aligned} \zeta _{\mathfrak {so}(5)}(s) := \sum _\varphi \frac{1}{\dim (\varphi )^{s}} = 6^s\sum _{n,m\ge 1}\frac{1}{ m^{s}n^{s}(m+n)^{s}(m+2n)^{s}}, \end{aligned}$$

where the $\varphi$ are running through the finite-dimensional irreducible representations of $\mathfrak {so}(5)$. We prove the following; for the more precise statement see Theorem 5.14.

#### Theorem 1.2

The function $\zeta _{{\mathfrak {so}{(}}5)}$ has a meromorphic continuation to ${\mathbb {C}}$ whose positive poles are simple and occur for $s\in \{\frac{1}{2},\frac{1}{3}\}$.

It is well-known that the finite-dimensional representations of $\mathfrak {so}(5)$ can be doubly indexed as $(\varphi _{j,k})_{j,k\in {\mathbb {N}}}$ with $\dim (\varphi _{j,k})=\frac{1}{6}jk(j+k)(j+2k)$, which explains the last equality in (1.7). A general *n*-dimensional representation decomposes as a sum of these $\varphi _{j,k}$, each with some multiplicity. Therefore, as in the case $\mathfrak {su}(3)$, we find that

$$\begin{aligned} \sum _{n \ge 0} r_{\mathfrak {so}(5)}(n)q^n = \prod _{j,k \ge 1} \frac{1}{1-q^{\frac{jk(j+k)(j+2k)}{6}}}. \end{aligned}$$

We prove the following.

#### Theorem 1.3

As $n\rightarrow \infty$, we have, for any $N\in {\mathbb {N}}$,

$$\begin{aligned} r_{\mathfrak {so}(5)}(n) = \frac{C}{n^\frac{7}{12}}\exp \left( A_1 n^{\frac{1}{3}} + A_2 n^{\frac{2}{9}} + A_3 n^{\frac{1}{9}} + A_4 \right) \left( 1 + \sum _{j=2}^{N+1} \frac{B_j}{n^{\frac{j-1}{9}}} + O_N\left( {n^{-\frac{N+1}{9}}}\right) \right) , \end{aligned}$$

where *C*, $A_1$, $A_2$, $A_3$, and $A_4$ are given in (5.17)–(5.19) and the $B_j$ can be calculated explicitly.

### Statement of results

The main goal of this paper is to prove asymptotic formulas for a general class of partition functions. To state it, let $f:{\mathbb {N}}\rightarrow {\mathbb {N}}_0$, set $\Lambda :={\mathbb {N}}\setminus f^{-1}(\{0\})$, and for $q=e^{-z}$ ($z\in {\mathbb {C}}$ with $\textrm{Re}(z) > 0$), define

1.8 $$\begin{aligned} G_f(z):=\sum _{n\ge 0} p_f(n)q^n = \prod _{n\ge 1} \frac{1}{\left( 1-q^n\right) ^{f(n)}},\qquad L_f(s) := \sum _{n\ge 1} \frac{f(n)}{n^s}. \end{aligned}$$

We require the following key properties of these objects:

1. All poles of $L_f$ are real. Let $\alpha >0$ be the largest pole of $L_f$. There exists $L\in {\mathbb {N}}$, such that for all primes *p*, we have $|\Lambda \setminus (p{\mathbb {N}}\cap \Lambda )|\ge L>\frac{\alpha }{2}$.
2. Condition (P2) is attached to $R\in {\mathbb {R}}^+$. The series $L_f(s)$ converges for some $s\in {\mathbb {C}}$, has a meromorphic continuation to $\{s\in {\mathbb {C}}:\textrm{Re}(s)\ge -R\}$, and is holomorphic on the line $\{s\in {\mathbb {C}}:\textrm{Re}(s)=-R\}$. The function $L_f^*(s):=\Gamma (s)\zeta (s+1)L_f(s)$ has only real poles $0<\alpha :=\gamma _1>\gamma _2>\dots$ that are all simple, except the possible pole at $s=0$, that may be double.
3. For some $a<\frac{\pi }{2}$, in every strip $\sigma _1\le \sigma \le \sigma _2$ in the domain of holomorphicity, we uniformly have, for $s=\sigma +it$,
  $$\begin{aligned} L_f(s) = O_{\sigma _1, \sigma _2}\left( e^{a|t|}\right) , \qquad |t| \rightarrow \infty . \end{aligned}$$

Note that (P1) implies that $|\Lambda \setminus (b{\mathbb {N}}\cap \Lambda )|\ge L>\frac{\alpha }{2}$ for all $b\ge 2$. The analytic properties of $L_f$ are a major ingredient needed to prove the following theorem, as analytic continuation in (P2) gives rise to asymptotic expansions of^6^$\textrm{Log}(G_f(z))$ and (P3) assists with vertical integration.

#### Theorem 1.4

Assume (P1) for $L\in {\mathbb {N}}$, (P2) for $R>0$, and (P3). Then, for some $M,N\in {\mathbb {N}}$,

$$\begin{aligned} p_f(n) = \frac{C}{n^b} \exp \left( A_1n^\frac{\alpha }{\alpha +1}+\sum _{j=2}^M A_jn^{\alpha _j}\right) \left( 1+\sum \limits _{j=2}^N \frac{B_j}{n^{\beta _j}} + O_{L,R}\left( n^{-\min \left\{ \frac{2L-\alpha }{2(\alpha +1)},\frac{R}{\alpha +1}\right\} }\right) \right) , \end{aligned}$$

where $0\le \alpha _M<\alpha _{M-1}<\cdots \alpha _2<\alpha _1=\frac{\alpha }{\alpha +1}$ are given by^7^${\mathcal {L}}$ (defined in (1.9)), and $0<\beta _2<\beta _3<\dots$ are given by ${\mathcal {M}}+{\mathcal {N}}$, where ${\mathcal {M}}$ and ${\mathcal {N}}$ are defined in (1.10) and (1.11), respectively. The coefficients $A_j$ and $B_j$ can be calculated explicitly; the constants $A_1$, *C*, and *b* are provided in (1.12) and (1.13). Moreover, if $\alpha$ is the only positive pole of $L_f$, then we have $M=1$.

#### Remarks

1. Debruyne and Tenenbaum [17] proved Theorem 1.4 in the special case that *f* is the indicator function of a subset $\Lambda$ of ${\mathbb {N}}$. They also assumed that the associated *L*-function can be analytically continued except for one pole in $0<\alpha \le 1$. In (P1), the assumption that $|\Lambda \setminus (p{\mathbb {N}}\cap \Lambda )|\ge L$ is used in Lemma 3.1 to bound minor arcs, whereas the additional assumption $L>\frac{\alpha }{2}$, that was automatically satisfied in [17], ensures that the bounds for the minor arcs are sufficient.
2. The complexity of the exponential term depends on the number and positions of the positive poles of $L_f$. Theorem 4.4 is more explicit and covers the case of exactly two positive poles. This case has importance for representation numbers of $\mathfrak {su}(3)$ and $\mathfrak {so}(5)$.

In Sect. 2, we collect some analytic tools, properties of special functions and useful properties of asymptotic expansions that are heavily used throughout the paper. In Sect. 3, we apply the Circle Method and calculate asymptotic expansions for the saddle point $\rho _n$ and the value of the generating function $G_f(\rho _n)$. In Sect. 4, we complete the proof of Theorem 1.4, and we also state and prove a more explicit version of Theorem 1.4 in the case that $L_f$ has two positive poles (Theorem 4.4). The proofs of Theorems 1.1, 1.2, and 1.3 are given in Sect. 5; this includes a detailed study of the Witten zeta function $\zeta _{\mathfrak {so}(5)}$ which is of independent interest.

## Notation

For $\beta \in {{\mathbb {R}}}$, we denote by $\{\beta \}:=\beta -\lfloor \beta \rfloor$ the *fractional part* of $\beta$. As usual, we set ${\mathbb {H}}:=\{\tau \in {\mathbb {C}}:\textrm{Im}(\tau )>0\}$ and ${\mathbb {E}}:=\{z\in {\mathbb {C}}:|z|<1\}$. For $\delta >0$, we define

$$\begin{aligned} {\mathcal {C}}_\delta := \left\{ z \in {\mathbb {C}}:|\textrm{Arg}(z)| \le \tfrac{\pi }{2} - \delta \right\} , \end{aligned}$$

where ${\text {Arg}}$ uses the principal branch of the complex argument. For $r>0$ and $z\in {\mathbb {C}}$, we set

$$\begin{aligned} B_r(z) := \{ w \in {{\mathbb {C}}}: |w - z| < r\}. \end{aligned}$$

For $a,b\in {\mathbb {R}}$, we let ${\mathcal {R}}_{a,b;K}$ be the rectangle with vertices $a\pm iK$ and $b\pm iK$, and we let $\partial {\mathcal {R}}_{a,b;K}$ be the path along the boundary of ${\mathcal {R}}_{a,b;K}$, surrounded once counterclockwise. For $-\infty \le a<b\le \infty$, we denote $S_{a,b}:=\{z\in {\mathbb {C}}:a<\textrm{Re}(z)<b\}$. We also set, for real $\sigma _1\le \sigma _2$ and $\delta >0$,

$$\begin{aligned} S_{\sigma _1,\sigma _2,\delta } := \{s\in {\mathbb {C}}: \sigma _1\le \textrm{Re}(s)\le \sigma _2\} \Bigg \backslash \left( B_\delta \left( \frac{1}{2}\right) \cup \bigcup _{j=-\infty }^1 B_\delta \left( \frac{j}{3}\right) \right) . \end{aligned}$$

For $k\in {\mathbb {N}}$ and $s\in {\mathbb {C}}$, the *falling factorial* is $(s)_k:=s(s-1)\ldots (s-k+1)$. For $f:{\mathbb {N}}\rightarrow {\mathbb {N}}_0$, we let ${\mathcal {P}}$ be the set of poles of $L^*_f$, and for $R>0$ we denote by ${\mathcal {P}}_R$ the union of the poles of $L_f^*$ greater than $-R$ with $\{0\}$. We define

1.9 $$\begin{aligned} {\mathcal {L}}&:= \frac{1}{\alpha +1}{\mathcal {P}}_R + \sum _{\mu \in {\mathcal {P}}_R} \left( \frac{\mu +1}{\alpha +1}-1\right) {\mathbb {N}}_0, \end{aligned}$$

1.10 $$\begin{aligned} {\mathcal {M}}&:= \frac{\alpha }{\alpha +1}{\mathbb {N}}_0 + \left( -\sum _{\mu \in {\mathcal {P}}_R} \left( \frac{\mu +1}{\alpha +1}-1\right) {\mathbb {N}}_0\right) \cap \left[ 0,\frac{R+\alpha }{\alpha +1}\right) , \end{aligned}$$

1.11 $$\begin{aligned} {\mathcal {N}}&:= \left\{ \sum _{j=1}^K b_j\theta _j : b_j,K\in {\mathbb {N}}_0,\theta _j\in (-{\mathcal {L}})\cap \left( 0,\frac{R}{\alpha +1}\right) \right\} . \end{aligned}$$

We set, with $\omega _\alpha :={\text {Res}}_{s=\alpha }L_f(s)$,

1.12 $$\begin{aligned} A_1&:= \left( 1+\frac{1}{\alpha }\right) (\omega _\alpha \Gamma (\alpha +1)\zeta (\alpha +1))^\frac{1}{\alpha +1},\nonumber \\ C&:= \frac{e^{L_f'(0)}(\omega _\alpha \Gamma (\alpha +1)\zeta (\alpha +1))^\frac{\frac{1}{2}-L_f(0)}{\alpha +1}}{\sqrt{2\pi (\alpha +1)}},\end{aligned}$$

1.13 $$\begin{aligned} b&:= \frac{1-L_f(0)+\frac{\alpha }{2}}{\alpha +1}. \end{aligned}$$

## Preliminaries

In this section, we collect and prove some tools used in this paper.

### Tools from complex analysis

We require the following results from complex analysis. The first theorem describes Taylor coefficients of the inverse of a biholomorphic function; for a proof, see Corollary 11.2 on p. 437 of [11].

#### Proposition 2.1

Let $\phi :B_r(0)\rightarrow D$ be a holomorphic function such that $\phi (0)=0$ and $\phi '(0)\ne 0$, with $\phi (z)=:\sum _{n\ge 1}a_nz^n$. Then $\phi$ is locally biholomorphic and its local inverse of $\phi$ has a power series expansion $\phi ^{-1}(w)=:\sum _{k\ge 1}b_kw^k$, where

$$\begin{aligned} b_k = \frac{1}{ka_1^{k}} \sum _{\begin{array}{c} \ell _1,\ell _2, \ell _3 ... \ge 0 \\ \ell _1 + 2\ell _2 + 3\ell _3 + \cdots = k-1 \end{array}} (-1)^{\ell _1 + \ell _2 +\ell _3+\cdots } \frac{k \ldots (k-1+\ell _1+\ell _2+\cdots )}{\ell _1! \ell _2! \ell _3! \ldots }\left( \frac{a_2}{a_1}\right) ^{\ell _1} \left( \frac{a_3}{a_1}\right) ^{\ell _2} \ldots . \end{aligned}$$

To deal with certain zeros of holomorphic functions, we require the following result from complex analysis, the proof of which is quickly obtained from Exercise 7.29 (i) in [10].

#### Proposition 2.2

Let $r>0$ and let $\phi _n:B_r(0)\rightarrow {\mathbb {C}}$ be a sequence of holomorphic functions that converges uniformly on compact sets to a holomorphic function $\phi :B_r(0)\rightarrow {\mathbb {C}}$, with $\phi '(0)\ne 0$. Then there exist $r>\kappa _1>0$ and $\kappa _2>0$ such that, for all *n* sufficiently large, the restrictions $\phi _n|_{B_{\kappa _1}(0)}:B_{\kappa _1}(0)\rightarrow \phi _n(B_{\kappa _1}(0))$ are biholomorphic and $B_{\kappa _2}(0)\subset \phi _n(B_{\kappa _1}(0))$. In particular, the restrictions $\phi _n^{-1}|_{B_{\kappa _2}(0)}:B_{\kappa _2}(0)\rightarrow \phi _n^{-1}(B_{\kappa _2}(0))$ are biholomorphic functions.

### Asymptotic expansions

We require two classes of asymptotic expansions.

#### Definition

Let $$R\in {\mathbb {R}}$$.

1. Let $g:{\mathbb {R}}^+\rightarrow {\mathbb {C}}$ be a function. Then $g\in {\mathcal {K}}(R)$ if there exist real numbers $\nu _{g,1}<\nu _{g,2}<\nu _{g,3}<\dots<\nu _{g,N}<R$ and complex numbers $a_{g,j}$ such that
  $$\begin{aligned} g(x) = \sum _{j=1}^{N_g} \frac{a_{g,j}}{x^{\nu _{g,j}}} + O_R\left( x^{-R}\right) ,\qquad (x\rightarrow \infty ). \end{aligned}$$
2. Let $\phi$ be holomorphic on the right half-plane. Then $\phi \in {\mathcal {H}}(R)$ if there are real numbers $\nu _{\phi ,1}<\nu _{\phi ,2}<\nu _{\phi ,3}<\dots<\nu _{\phi ,N}<R$ and $a_{\phi ,j}\in {\mathbb {C}}$ such that, for all $k\in {\mathbb {N}}_0$ and $0<\delta <\frac{\pi }{2}$,
  2.1 $$\begin{aligned} \phi ^{(k)}(z) = \sum _{j=1}^{N_\phi } (\nu _{\phi , j})_k a_{\phi ,j} z^{\nu _{\phi , j}-k} + O_{\delta , R, k}\left( |z|^{R-k} \right) , \qquad (z \rightarrow 0, z \in {\mathcal {C}}_\delta ). \end{aligned}$$

If there is no risk of confusion, then we write *N*, $\nu _j$, and $a_j$ in the above. The *R*-dependence of the error only matters if *R* varies, for instance, if we can choose it to be arbitrarily large.

Note that any sequence *g*(*n*) with

2.2 $$\begin{aligned} g(n) = \sum _{j=1}^N \frac{a_j}{n^{\nu _j}} + O_R\left( n^{-R}\right) ,\qquad (n\rightarrow \infty ), \end{aligned}$$

can be extended to a function *g* in ${\mathcal {K}}(R)$. Conversely, each function in ${\mathcal {K}}(R)$ can be restricted to a sequence $\{g(n)\}_{n\in {\mathbb {N}}}$ satisfying (2.2). In addition, we include functions in ${\mathcal {K}}(R)$ that have asymptotic expansion as in (1), but are initially defined only on intervals $(r,\infty )$ for some large $r>0$. The reason for this is that it does not matter how the function is defined up to *r*, and therefore it can always be continued to $(0,\infty )$. If $g\in {\mathcal {K}}(R)$ for all $R>0$, then we write

2.3 $$\begin{aligned} g(x) = \sum _{j\ge 1} \frac{a_j}{x^{\nu _j}},\qquad (x\rightarrow \infty ). \end{aligned}$$

We use the same abbreviation if $\phi \in {\mathcal {H}}(R)$ for all $R>0$. In this case we write $g\in {\mathcal {K}}(\infty )$ and $\phi \in {\mathcal {H}}(\infty )$, respectively. In some situations, we write for $R\in {\mathbb {R}}\cup \{\infty \}$

$$\begin{aligned} g(x) = \sum _{j=1}^{N} \frac{a_{g,j}}{x^{\nu _{g,j}}} + O_R\left( x^{-R} \right) , \end{aligned}$$

where *R* might depend on the choice of the function *g*. If $R=\infty$, then one may ignore the error $O_R(x^{-R})$ and use the notation (2.3) instead. We have the following useful lemmas, that can be obtained by a straightforward calculation.

#### Lemma 2.3

Let $R_1, R_2 \in {\mathbb {R}}$, $\lambda \in {\mathbb {C}}$, $g \in {\mathcal {K}}(R_1)$, and $h \in {\mathcal {K}}(R_2)$. Then we have the following:

1. We have $\lambda g\in {\mathcal {K}}(R_1)$ and $g+h\in {\mathcal {K}}(\min \{R_1,R_2\})$. The exponents $\nu _{g+h,j}$ run through
  $$\begin{aligned} (\{ \nu _{g,j} :1 \le j \le N_g \} \cup \{ \nu _{h,j} :1 \le j \le N_h \}) \cap (-\infty , \min \{R_1, R_2\}). \end{aligned}$$
2. We have $gh\in {\mathcal {K}}(\min \{R_1+\nu _{h,1},R_2+\nu _{g,1}\})$. The exponents $\nu _{gh,j}$ run through
  $$\begin{aligned} (\{ \nu _{g,j} :1 \le j \le N_g \} + \{ \nu _{h,j} :1 \le j \le N_h \}) \cap (-\infty , \min \{ R_1 + \nu _{h,1}, R_2 + \nu _{g,1}\}). \end{aligned}$$

We next deal with compositions of asymptotic expansions with holomorphic functions.

#### Lemma 2.4

Let $0<R\le \infty$, $g\in {\mathcal {K}}(R)$ with $\nu _{g,1}=0$ and *h* holomorphic at $a_{g,1}$. Then $(h\circ g)(x)$ is defined for all $x>0$ sufficiently large, and we have $h\circ g\in {\mathcal {K}}(R)$ with

$$\begin{aligned} \{\nu _{h\circ g,j} : 1\le j\le N_{h\circ g}\} = \left( \sum _{j=1}^{N_g}\nu _{g,j}{\mathbb {N}}_0\right) \cap [0,R). \end{aligned}$$

We need a similar result for general asymptotic expansions.

#### Lemma 2.5

Let $0<R_1,R_2\le \infty$, $\phi \in {\mathcal {H}}(R_1)$, $g\in {\mathcal {K}}(R_2)$, and $R:=\min \{R_2-\nu _{g,1},\nu _{g,1}R_1\}$. Assume $\nu _{g,1}>0$ and $g(x)>0$ for *x* sufficiently large. Then $\phi \circ g\in {\mathcal {K}}(R)$, $a_{\phi \circ g,1}=a_{\phi ,1}a_{g,1}^{\nu _{\phi ,1}}$, and

$$\begin{aligned} \{ \nu _{\phi \circ g,j} :1 \le j \le N_{\phi \circ g}\} = \left( \nu _{g,1} \{ \nu _{\phi , 1}, ..., \nu _{\phi , N_\phi }\} + \sum _{j=2}^{N_{g}} (\nu _{g,j} - \nu _{g,1}) {\mathbb {N}}_0\right) \cap (-\infty , R). \end{aligned}$$

### Special functions

The following theorem collects some facts about the Gamma function.

#### Proposition 2.6

(See [1, 35]) Let $\gamma$ denote the Euler–Mascheroni constant.

1. The gamma function $\Gamma$ is holomorphic on ${\mathbb {C}}\setminus (-{\mathbb {N}}_0)$ with simple poles in $-{\mathbb {N}}_0$. For $n\in {\mathbb {N}}_0$ we have ${\text {Res}}_{s=-n}\Gamma (s)=\frac{(-1)^n}{n!}$.
2. For $s = \sigma + it \in {\mathbb {C}}$ with $\sigma \in I$ for a compact interval $I \subset [\frac{1}{2}, \infty )$, we uniformly have
  $$\begin{aligned} \max \left\{ 1, |t|^{\sigma - \frac{1}{2}}\right\} e^{- \frac{\pi |t|}{2} } \ll _I |\Gamma (s)| \ll _I \max \left\{ 1, |t|^{\sigma - \frac{1}{2}}\right\} e^{- \frac{\pi |t|}{2} }. \end{aligned}$$
  The bound also holds for compact intervals $I \subset {{\mathbb {R}}}$ if $|t| \ge 1$.
3. Near $s=0$, we have the Laurent series expansion $\Gamma (s) = \frac{1}{s} - \gamma + O(s)$.
4. For all $s \in {\mathbb {C}}\setminus {\mathbb {Z}}$, we have $\Gamma (s)\Gamma (1-s) = \frac{\pi }{\sin (\pi s)}$.

For $s,z \in {{\mathbb {C}}}$ with $s \notin -{\mathbb {N}}$, the *generalized Binomial coefficient* is defined by

$$\begin{aligned} \left( {\begin{array}{c}s\\ z\end{array}}\right) := \frac{\Gamma (s+1)}{\Gamma (z+1)\Gamma (s-z+1)}. \end{aligned}$$

We require the following properties of the Riemann zeta function.

#### Proposition 2.7

(See [2, 9, 35])

1. The $\zeta$-function has a meromorphic continuation to ${{\mathbb {C}}}$ with only a simple pole at $s=1$ with residue 1. For $s \in {{\mathbb {C}}}$ we have (as identity between meromorphic functions)
  $$\begin{aligned} \zeta (s) = 2^s \pi ^{s-1} \sin \left( \frac{\pi s}{2}\right) \Gamma (1-s) \zeta (1-s). \end{aligned}$$
2. For $I := [\sigma _0, \sigma _1]$ and $s = \sigma +it \in {\mathbb {C}}$, there exists $m_I\in {\mathbb {Z}}$, such that for $\sigma \in I$
  $$\begin{aligned} \zeta (s) \ll (1+|t|)^{m_I},\qquad (|t|\rightarrow \infty ). \end{aligned}$$
3. Near $s=1$, we have the Laurent series expansion $\zeta (s) = \frac{1}{s-1}+\gamma + O(s-1)$.

For the Saddle Point Method we need the following estimate.

#### Lemma 2.8

Let $\mu _n$ be an increasing unbounded sequence of positive real numbers, $B>0$, and *P* a polynomial of degree $m\in {\mathbb {N}}_0$. Then we have

$$\begin{aligned} \int _{-\mu _n}^{\mu _n} P(x) e^{-Bx^2} dx = \int _{-\infty }^{\infty } P(x) e^{-Bx^2} dx + O_{B,P}\left( \mu _n^{\frac{m-1}{2}} e^{-B\mu _n^2} \right) . \end{aligned}$$

Finally, we require the following in our study of the Witten zeta function $\zeta _{{\mathfrak {so}{(}}5)}.$

#### Lemma 2.9

Let $n\in {\mathbb {N}}_0$. The function $g:{\mathbb {R}}\rightarrow {\mathbb {R}}$ defined as $g(u):=e^{|u|}\int _{-\infty }^\infty |v|^ne^{-|v|-|v+u|}dv$ satisfies $g(u)=O_n(u^{n+1})$ as $|u|\rightarrow \infty$.

#### Proof

Let $u\ge 0$. Then we have

$$\begin{aligned} g(u) = \frac{n!}{2^{n+1}}\sum _{j=0}^n \frac{2^j}{j!}u^j + \frac{u^{n+1}}{n+1} + \frac{n!}{2^{n+1}} = O_n\left( u^{n+1}\right) . \end{aligned}$$

The lemma follows, since *g* is an even function. $\square$

## Minor and major arcs

### The minor arcs

For $z \in {\mathbb {C}}$ with ${\text {Re}}(z) > 0$, we define, with $G_f$ given in (1.8),

$$\begin{aligned} \Phi _f(z):=\textrm{Log}(G_f(z)). \end{aligned}$$

Note that we assume throughout, that the function *f* grows polynomially, which is implicitly part of (P2). We apply Cauchy’s Theorem, writing

$$\begin{aligned} p_f(n) = \frac{1}{2\pi } \int _{-\pi }^{\pi } \exp \left( n(\varrho _n + it) + \Phi _f(\varrho _n + it)\right) dt, \end{aligned}$$

where $\varrho _n \rightarrow 0^+$ is determined in Sect. 3.3. We split the integral into two parts, the major and minor arcs, for any $\beta \ge 1$

3.1 $$\begin{aligned} p_f(n)&= \frac{e^{\varrho _n n}}{2\pi }\int _{|t| \le \varrho _n^\beta } \exp \left( int + \Phi _f(\varrho _n + it)\right) dt \nonumber \\&\quad + \frac{e^{\varrho _n n}}{2\pi } \int _{\varrho ^\beta \le |t| \le \pi } \exp \left( int + \Phi _f(\varrho _n + it)\right) dt. \end{aligned}$$

The first integral provides the main terms in the asymptotic expansion for $p_f(n)$, the second integral is negligible, as the following lemma shows.

#### Lemma 3.1

Let $1<\beta <1+\frac{\alpha }{2}$ and assume that *f* satisfies the conditions of Theorem 1.4. Then

$$\begin{aligned} \int _{\frac{\varrho _n^\beta }{2\pi } \le |t| \le \frac{1}{2}} e^{2\pi int} G_f(\varrho _n + 2 \pi i t) dt \ {\ll _L \varrho _n^{L+1} G_f(\varrho _n)}. \end{aligned}$$

#### Sketch of proof

The proof may be adapted from [17, Lemma 3.1]. That is, we estimate the quotient,

$$\begin{aligned} \frac{|G_f(\rho _n+2\pi it)|}{G_f(\rho _n)} \le \prod _{m\ge 1} \left( 1+\frac{16||mt||^2}{e^{m\rho _n}m^2\rho _n^2}\right) ^{-\frac{f(m)}{2}}, \end{aligned}$$

where ||*x*|| is the distance from *x* to the nearest integer. We then throw away *m*-th factors depending on the location of $t\in [\frac{\rho _n^\beta }{2\pi },\frac{1}{2}]$. The proof follows [17, Lemma 3.1] *mutatis mutandis*; key facts are hypotheses (P1) and (P3) of Theorem 1.4 and that (which follows from [35, Theorem 7.28 (1)])

$$\begin{aligned} \sum _{1 \le m \le x} f(m) \sim \frac{\textrm{Res}_{s=\alpha } L_f}{\alpha } x^{\alpha }. \end{aligned}$$

$$\square $$

### Inverse Mellin transforms for generating functions

We start this subsection with a lemma on the asymptotic behavior of the function $\Phi _f$ near $z=0$.

#### Lemma 3.2

Let $f:{\mathbb {N}}\rightarrow {\mathbb {N}}_0$ satisfy (P2) with $R>0$ and (P3). Fix some $0<\delta <\frac{\pi }{2}-a$. Then we have, as $z\rightarrow 0$ in $C_\delta$,

$$\begin{aligned} \Phi _f(z) = \sum _{\begin{array}{c} \nu \in -{\mathcal {P}}_R\setminus \{0\} \end{array}} {\text {Res}}_{s=-\nu } L_f^*(s)z^{\nu } - L_f(0)\textrm{Log}(z) + L_f'(0) + O_{R}\left( |z|^R\right) . \end{aligned}$$

For the *k*-th derivative ($k\in {\mathbb {N}}$), we have

$$\begin{aligned} \Phi _f^{(k)}(z) = \sum _{\begin{array}{c} \nu \in -{\mathcal {P}}_R\setminus \{0\} \end{array}} (\nu )_k {\text {Res}}_{s=-\nu } L_f^*(s)z^{\nu - k} + \frac{(-1)^k(k-1)! L_f(0)}{z^k} + O_{R,k}\left( |z|^{R-k}\right) . \end{aligned}$$

#### Proof

With $J_f(s;z):=L_f^*(s)z^{-s}$, we obtain, for $\kappa \in {\mathbb {N}}_0$,

3.2 $$\begin{aligned}&2\pi i\Phi _f^{(\kappa )}(z) \nonumber \\&\quad = \frac{d^\kappa }{dz^\kappa }\left( \int _{-R-i\infty }^{-R+i\infty } + \lim _{K\rightarrow \infty } \left( \int _{ \partial {\mathcal {R}}_{-R,\alpha +1;K}} + \int _{\alpha +1-iK}^{-R-iK} + \int _{-R+iK}^{\alpha +1+iK}\hspace{.1cm}\right) \right) J_f(s;z) ds. \end{aligned}$$

Here we use (P2), giving that there are no poles of $J_f(s;z)$ on the path of integration. By Proposition 2.7 (2), [8, Theorem. 2.1 (3)], and (P3), we find a constant $c(R,\kappa )$ such that, as $|v|\rightarrow \infty$,

$$\begin{aligned} \left| L_f^*(-R+iv)\right| \ll _{R} (1+|v|)^{c(R,\kappa )} e^{-\left( \frac{\pi }{2}-a\right) |v|}. \end{aligned}$$

This yields, with Leibniz’s integral rule and $0<\delta <\frac{\pi }{2}-a$,

$$\begin{aligned} \left| \frac{d^\kappa }{dz^\kappa }\int _{-R-i\infty }^{-R+i\infty } J_f(s;z) ds\right| \ll _{R,\kappa } |z|^{R-\kappa }. \end{aligned}$$

For the second integral in (3.2), applying the Residue Theorem gives

$$\begin{aligned}{} & {} \frac{d^\kappa }{dz^\kappa }\lim _{K\rightarrow \infty }\frac{1}{2\pi i}\int _{ \partial {\mathcal {R}}_{-R,\alpha +1;K}} J_f(s;z) ds\\{} & {} \quad = \sum _{\begin{array}{c} \nu \in -{\mathcal {P}}_R\setminus \{0\} \end{array}} (\nu )_\kappa {\text {Res}}_{s=-\nu } L_f^*(s) z^{\nu -\kappa } + \frac{d^\kappa }{dz^\kappa }\left( -L_f(0)\textrm{Log}(z)+L_f'(0)\right) , \end{aligned}$$

since $s=0$ is a double pole of $J_f(s;z)$. For the last two integrals in (3.2) we have, for some ${m(I)}\in {\mathbb {N}}_0$, depending on $I:={[-R,\alpha +1]}$,

$$\begin{aligned} \left| \int _{-R\pm iK}^{\alpha +1\pm iK} J_f(s;z) ds\right| \ll _I (1+|K|)^{m(I)}\max \left\{ |z|^{\alpha +1},|z|^{-R}\right\} e^{-(\delta -a)|K|}, \end{aligned}$$

which vanishes as $K\rightarrow \infty$ and thus the claim follows by distinguishing $\kappa =0$ and $\kappa \in {\mathbb {N}}$. $\square$

### Approximation of saddle points

We now approximately solve the saddle point equations

3.3 $$\begin{aligned} -\Phi '_f(\varrho ) = n= - \Phi '_f(\varrho _n). \end{aligned}$$

The following proposition provides an asymptotic formula for certain functions.

#### Proposition 3.3

Let $\phi \in {\mathcal {H}}(R)$ with $R>0$, $\nu _{\phi ,1}<0$, and $a_{\phi ,1}>0$. Assume that $\phi ({\mathbb {R}}^+)\subset {\mathbb {R}}$.

Then we have the following:

1. There exists a positive sequence $(\rho _n)_{n\in {\mathbb {N}}}$, such that for all *n* sufficiently large, $\phi (\rho _n)=n$ holds.
2. We have^8^$\rho \in {\mathcal {K}}(1-\frac{R+1}{\nu _{\phi ,1}})$, $a_{\rho ,1}=a_{\phi ,1}^{-\frac{1}{\nu _{\phi ,1}}}$, and the corresponding exponent set
  $$\begin{aligned} \{\nu _{\rho ,j} : 1\le j\le N_\rho \} = \left( -\frac{1}{\nu _{\phi ,1}}+\sum _{j=1}^{N_\phi } \left( 1-\frac{\nu _{\phi ,j}}{\nu _{\phi ,1}}\right) {\mathbb {N}}_0\right) \cap \left( -\infty ,1-\frac{R+1}{\nu _{\phi ,1}}\right) . \end{aligned}$$
  In particular, we have $\rho _n \rightarrow 0^+$.

#### Proof

In the proof we abbreviate $\nu _n:=\nu _{\phi ,n}$ and $a_n:=a_{\phi ,n}$.

(1) For $$n\in {\mathbb {N}}$$, set

$$\begin{aligned} \psi _n(w) := -1 + \frac{1}{n}\phi \left( \left( \frac{n}{a_1}\right) ^\frac{1}{\nu _1}w\right) . \end{aligned}$$

As $\phi$ is holomorphic on the right-half plane by assumption, so are the $\psi _n$. Using (2.1), write

3.4 $$\begin{aligned} \psi _n(w) = w^{\nu _1} -1 + E_n(w), \end{aligned}$$

where the error satisfies

$$\begin{aligned} E_n(w) = \frac{1}{n}\sum _{\begin{array}{c} j=2 \end{array}}^{N_\phi } a_j\left( \frac{n}{a_1}\right) ^\frac{\nu _j}{\nu _1}w^{\nu _j} + O_R\left( n^{\frac{R}{\nu _1}-1}|w|^R\right) . \end{aligned}$$

We next show that, for all *n* sufficiently large, the $\psi _n$ only have one zero near $w=1$. We argue with Rouché’s Theorem. First, we find that, for *n* sufficiently large, the inequality

3.5 $$\begin{aligned} |E_n(w)| < \left| 1 - w^{\nu _1}\right| + \left| w^{\nu _1} - 1 + E_n(w) \right| = \left| 1 - w^{\nu _1}\right| + |\psi _n(w)| \end{aligned}$$

holds on the entire boundary of $B_{\kappa (\nu _1)}(1)$, with $0<\kappa (\nu _1)<\frac{1}{2}$ sufficiently small such that $w\mapsto 1-w^{\nu _1}$ only has one zero in $B_{\kappa (\nu _1)}(1)$. By Rouché’s Theorem and (3.5), for *n* sufficiently large $\psi _n$ also has exactly one zero in $B_{\kappa (\nu _1)}(1)$. We denote this zero of $\psi _n$ by $w_n$. It is real as $\phi$ is real-valued on the positive real line and a holomorphic function. One can show that $\rho _n=(\frac{n}{a_1})^\frac{1}{\nu _1}w_n>0$ satisfies $\phi (\rho _n)=n$.

(2) We first give an expansion for $w_n$. By Proposition 2.2, there exists $\kappa >0$, such that for all *n* sufficiently large and all $z\in B_\kappa (0)$, the inverse functions $\psi _n^{-1}$ of $\psi _n$ are defined and holomorphic in $B_\kappa (1)$. Using this, we can calculate $w_n$, satisfying $\psi _n(w_n)=0$. For this, let

$$\begin{aligned} h_n(w):=\psi _n(w+1)-\psi _n(1). \end{aligned}$$

We have $h_n(0)=0$, and we find, with Proposition 2.1,

$$\begin{aligned} w_n - 1 = h_n^{-1}(-\psi _n(1)) = \sum _{m \ge 1} (-1)^mb_m(n)\psi _n(1)^m, \end{aligned}$$

where the $b_m$ can be explicitly calculated. First, $\psi _n(1)^m$ ($m\in {\mathbb {N}}_0$) have expansions in *n* by (3.4) and Lemma 2.4. They have exponent set $\sum _{2\le j\le N_\phi }(1-\frac{\nu _j}{\nu _1}){\mathbb {N}}_0\cap [0,1-\frac{R}{\nu _1})$. We find, for $k\in {\mathbb {N}}$,

3.6 $$\begin{aligned} \psi _n^{(k)}(1) = \frac{1}{n}\sum _{j=1}^{N_\phi } (\nu _j)_ka_j\left( \frac{n}{a_1}\right) ^\frac{\nu _j}{\nu _1} + O_R\left( n^{\frac{R}{\nu _1}-1}\right) . \end{aligned}$$

Again by Lemma 2.4, and (3.6), $\psi ^{(k)}_n(1)$ ($k\in {\mathbb {N}}_0$) has expansions in *n*, with exponent set $(\sum _{2\le j\le N_\phi }(1-\frac{\nu _j}{\nu _1}){\mathbb {N}}_0)\cap [0,1-\frac{R}{\nu _1})$. By Lemma 2.4 we have the following expansion in *n*

$$\begin{aligned} \psi _n'(1)^{-m} = \left( \nu _1+\frac{1}{n}\sum _{j=2}^{N_\phi } \nu _ja_j\left( \frac{n}{a_1}\right) ^\frac{\nu _j}{\nu _1} + O_R\left( n^{\frac{R}{\nu _1}-1}\right) \right) ^{-m} \end{aligned}$$

with exponent set $(\sum _{2\le j\le N_\phi }(1-\frac{\nu _j}{\nu _1}){\mathbb {N}}_0)\cap [0,1-\frac{R}{\nu _1})$. By the formula in Proposition 2.1, the $b_m(n)$ are essentially sums and products of terms $\psi _n'(1)^{-1}$ and $\psi ^{(k)}_n(1)$, where $k\ge 2$. Hence, $b_m(n)$ has an expansion in *n*, with exponent set $(\sum _{2\le j\le N_\phi }(1-\frac{\nu _j}{\nu _1}){\mathbb {N}}_0)\cap [0,1-\frac{R}{\nu _1})$, and according to Lemma 2.3, the same holds for finite linear combinations $\sum _{1\le m\le M}(-1)^mb_m(n)\psi _n(1)^m$. As $\psi _n(1)=O(n^{\frac{\nu _2}{\nu _1}-1})$ for $n\rightarrow \infty$, one has, for *M* sufficiently large and not depending on *n*,

$$\begin{aligned} \sum _{m\ge M+1} (-1)^mb_m(n)\psi _n(1)^m = O_R\left( n^{\frac{R}{\nu _1}-1}\right) . \end{aligned}$$

Now, as $w_n\sim 1$, we conclude the theorem recalling that $\rho _n=(\frac{n}{a_1})^\frac{1}{\nu _1}w_n$. $\square$

We next apply Proposition 3.3 to $-\Phi _f'$. For the proof one may use Lemma 3.2 with $k=1$.

#### Corollary 3.4

Let $\rho _n$ solve (3.3). Assume that $f:{\mathbb {N}}\rightarrow {\mathbb {N}}_0$ satisfies the conditions of Theorem 1.4. Then $\rho \in {\mathcal {K}}(\frac{R}{\alpha +1}+1)$ with $a_{\rho ,1}=a_{-\Phi _f',1}^\frac{1}{\alpha +1} = (\omega _\alpha \Gamma (\alpha +1)\zeta (\alpha +1))^{\frac{1}{\alpha +1}}$ and we have

$$\begin{aligned} \{\nu _{\rho ,j}:1\le j\le N_\rho \} =\left( \!\frac{1}{\alpha +1} - \sum _{\begin{array}{c} \mu \in {{\mathcal {P}}_R} \end{array}}\left( \! \frac{\mu +1}{\alpha +1} - 1 \!\right) {\mathbb {N}}_0\right) \cap \left[ \!\frac{1}{\alpha + 1},\frac{R}{\alpha + 1} + 1\!\right) . \end{aligned}$$

### The major arcs

In this subsection, we approximate, for some $1+\frac{\alpha }{3}<\beta <1+\frac{\alpha }{2}$,

$$\begin{aligned} I_n := \int _{|t|\le \rho _n^\beta } \exp (\Phi _f(\rho _n+it)+int) dt, \end{aligned}$$

where $\alpha$ is the largest positive pole of $L_f$. The following lemma can be shown using [17, §4].

#### Lemma 3.5

Let $f:{\mathbb {N}}\rightarrow {\mathbb {N}}_0$ satisfy the conditions of Theorem 1.4, $\rho _n$ solve (3.3), and $N\in {\mathbb {N}}$. Then we have

$$\begin{aligned} I_n = \sqrt{2\pi } G_f(\varrho _n) \left( \frac{1}{\sqrt{\Phi ''_f(\varrho _n)}} + \sum _{2 \le k \le \frac{3H(N+\alpha )}{2\alpha }} \frac{(2k)! \lambda _{2k}(\varrho _n)}{2^k k! \Phi ''_f(\varrho _n)^{k+\frac{1}{2}}} + O_N\left( \varrho _n^N \right) \right) , \end{aligned}$$

where $$H:=\lceil \frac{N}{3(\beta -1-\frac{\alpha }{3})}\rceil +1$$ and

$$\begin{aligned} \lambda _{2k}(\varrho ) := (-1)^k \sum _{h=1}^{H} \frac{1}{h!} \sum _{\begin{array}{c} 3 \le m_1, ..., m_h \le \frac{3(N+\alpha )}{\alpha } \\ m_1 + \cdots + m_h = 2k \end{array}} \prod _{j=1}^h \frac{\Phi _f^{(m_j)}(\varrho )}{m_j!}. \end{aligned}$$

The following lemma shows that the first term in Lemma 3.5 dominates the others; its proof follows with Lemmas 2.5,  3.2, and Corollary 3.4 by a straightforward calculation.

#### Lemma 3.6

Let $k\ge 2$ and assume the conditions as in Lemma 3.5. Then we have

$$\begin{aligned} \frac{\lambda _{2k}(\rho _n)}{\Phi ''_f(\rho _n)^{k+\frac{1}{2}}} = \sum _{j=1}^M \frac{b_j}{n^{\eta _j}} + O_R\left( n^{-{R+1+\left( k-\left\lfloor \frac{2k}{3}\right\rfloor +\frac{3}{2}\right) }\frac{\alpha }{\alpha +1}}\right) , \end{aligned}$$

where the $\eta _j$ run through

$$\begin{aligned} \frac{\alpha +2}{2(\alpha +1)} + \frac{\alpha }{\alpha +1}{\mathbb {N}}_0 + \left( -\sum _{\mu \in {\mathcal {P}}_R} \left( \frac{\mu +1}{\alpha +1}-1\right) {\mathbb {N}}_0\right) \cap \left[ 0,\frac{R+\alpha }{\alpha +1}\right) . \end{aligned}$$

We next use Lemma 2.5 and Corollary 3.4 to give an asymptotic expansion for $G_f(\rho _n)$.

#### Lemma 3.7

Assume that $f:{\mathbb {N}}\rightarrow {\mathbb {N}}_0$ satisfies the conditions of Theorem 1.4. Then, we have

$$\begin{aligned} G_f(\varrho _n)&= \frac{e^{L_f'(0)}n^{\frac{L_f(0)}{\alpha +1}}}{a_{-\Phi _f',1}^{\frac{L_f(0)}{\alpha +1}}} \exp \left( \frac{1}{\alpha }(\omega _\alpha \Gamma (\alpha +1)\zeta (\alpha +1))^{\frac{1}{\alpha +1}} n^{\frac{\alpha }{\alpha +1}} + \sum _{j=2}^M C_j n^{\beta _j}\right) \\&\quad \times \left( 1 + \sum _{j = 1}^N \frac{B_j}{n^{\delta _j}} + O_R\left( n^{-\frac{R}{\alpha +1}}\right) \right) , \end{aligned}$$

where $0\le \beta _M<\dots<\beta _2<\frac{\alpha }{\alpha +1}$ run through ${\mathcal {L}}$ and $0<\delta _1<\delta _2<\dots <\delta _N$ through ${\mathcal {M}}+{\mathcal {N}}$.

#### Proof

Let $\phi (z):=\Phi _f(z)+L_f(0)\textrm{Log}(z)$ and $F:=\phi \circ \rho$. By Lemma 3.2, Proposition 3.3, and Lemma 2.5 we find that

3.7 $$\begin{aligned} \Phi _f(\varrho _n) + L_f(0)\log (\varrho _n) = L'_f(0) + \sum _{j = 1}^{N_F} \frac{a_{F,j}}{ n^{\nu _{F,j}}} + O_R\left( n^{-\frac{R}{\alpha +1}}\right) , \end{aligned}$$

where $\nu _{F,j}$ run through (the inclusion follows by Corollary 3.4)

3.8 $$\begin{aligned}&\left( -\frac{1}{\alpha +1}{\mathcal {P}}_R + \sum _{j=2}^{N_\rho } \left( \nu _{\rho ,j}-\frac{1}{\alpha +1}\right) {\mathbb {N}}_0\right) \cap \left( -\infty , \frac{R}{\alpha +1}\right) \nonumber \\&\quad \subset \left( -\frac{1}{\alpha +1}{\mathcal {P}}_R - \sum \limits _{\mu \in {\mathcal {P}}_R}\left( \frac{\mu +1}{\alpha +1} - 1\right) {\mathbb {N}}_0\right) \cap \left( -\infty , \frac{R}{\alpha +1}\right) . \end{aligned}$$

Note that, again by Lemmas 2.5 and 3.2, we obtain

$$\begin{aligned} a_{F,1} = a_{\phi ,1} a_{\rho ,1}^{\nu _{\phi ,1}} = \frac{1}{\alpha } (\omega _\alpha \Gamma (\alpha +1) \zeta (\alpha +1))^{\frac{1}{\alpha +1}}. \end{aligned}$$

We split the sum in (3.7) into two parts: one with nonpositive $\nu _{F,1},\dots ,\nu _{F,M}$, say, and the one with positive $\nu _{F,j}<\frac{R}{\alpha +1}$. Note that *M* is bounded and independent of *R*. Exponentiating (3.7) yields

$$\begin{aligned} \exp (\Phi _f(\rho _n)) = \rho _n^{-L_f(0)}e^{L_f'(0)}\exp \left( \sum _{j=M+1}^{N_{F}} \frac{a_{F,j}}{n^{\nu _{F,j}}} + O_R\left( n^{-\frac{R}{\alpha +1}}\right) \right) \exp \left( \sum _{j=1}^M \frac{a_{F,j}}{n^{\nu _{F,j}}}\right) . \end{aligned}$$

Note that the positive $\nu _{F,j}$ run through (3.8) with $-\infty$ replaced by 0. By Lemma 2.4, we have

$$\begin{aligned} \exp \left( \sum _{j=M+1}^{N_F} \frac{a_{F,j}}{n^{\nu _{F,j}}} + O_R\left( n^{-\frac{R}{\alpha +1}}\right) \right) = 1 + \sum _{j=1}^{K} \frac{H_j}{n^{\varepsilon _j}} + O_R\left( n^{-\frac{R}{\alpha +1}}\right) \end{aligned}$$

for some $K\in {\mathbb {N}}$ and with exponents $\varepsilon _j$ running through ${\mathcal {N}}$. Recall that, by Corollary 3.4, we have $\rho _n\sim a_{\rho ,1}n^{-\frac{1}{\alpha +1}}$. Now set $h(n):=n^{-\frac{L_f(0)}{\alpha +1}}\rho _n^{-L_f(0)}$. A straightforward calculation using Corollary 3.4 shows that $h\in {\mathcal {K}}(\frac{R+\alpha }{\alpha +1})$ with exponent set $(-\sum _{\mu \in {\mathcal {P}}_R}(\frac{\mu +1}{\alpha +1}-1){\mathbb {N}}_0)\cap [0,\frac{R+\alpha }{\alpha +1})\subset {\mathcal {M}}$ and $a_{h,1}=a_{-\Phi _f',1}^{-\frac{L_f(0)}{\alpha +1}}$. By Lemma 2.3 (2), we obtain, for some $N\in {\mathbb {N}}$, $B_j\in {\mathbb {C}}$, and $\delta _j$ running through ${\mathcal {M}}+{\mathcal {N}}$,

$$\begin{aligned} h(n)\left( 1+\sum _{j=1}^K \frac{H_j}{n^{\varepsilon _j}}+O_R\left( n^{-\frac{R}{\alpha +1}}\right) \right) = a_{h,1}\left( 1+\sum _{j=1}^N \frac{B_j}{n^{\delta _j}}+O_R\left( n^{-\frac{R}{\alpha +1}}\right) \right) . \end{aligned}$$

Setting $C_j:=a_{F,j}$ for $1\le j\le M$, the lemma follows. $\square$

Another important step for the proof of our main theorem is the following lemma.

#### Lemma 3.8

Let $f:{\mathbb {N}}\rightarrow {\mathbb {N}}_0$ satisfy the conditions of Theorem 1.4. Then we have, as $n\rightarrow \infty$,

$$\begin{aligned} e^{n\rho _n}&= \exp \left( (\omega _\alpha \Gamma (\alpha +1)\zeta (\alpha +1))^\frac{1}{\alpha +1}n^{\frac{\alpha }{\alpha +1}}+\sum \limits _{j=2}^{M} a_{\rho ,j}n^{\eta _{j}} \right) \\&\quad \times \left( 1 + \sum _{j=1}^N \frac{D_j}{n^{\mu _j}} + O_R\left( n^{-\frac{R}{\alpha +1}}\right) \right) \end{aligned}$$

for some $1\le M\le N_\rho$, with $\frac{\alpha }{\alpha +1}>\eta _2>\dots >\eta _M\ge 0$ running through ${\mathcal {L}}$ and the $\mu _j$ through ${\mathcal {N}}$.

#### Proof

Let $g(n):=n\rho _n$. By Corollary 3.4 we have $g\in {\mathcal {K}}(\frac{R}{\alpha +1})$ with exponent set

$$\begin{aligned} \{\nu _{g,j} : 1\le j\le N_\rho \}= & {} \left( -1+\frac{1}{\alpha +1}-\sum _{\begin{array}{c} \mu \in {\mathcal {P}}_R \end{array}} \left( \frac{\mu +1}{\alpha +1}-1\right) {\mathbb {N}}_0\right) \\{} & {} \cap \left[ -1+\frac{1}{\alpha +1},\frac{R}{\alpha +1}\right) . \end{aligned}$$

Hence, for some $1\le M\le N_\rho$, we obtain

$$\begin{aligned} e^{n\rho _n} = \exp \left( a_{-\Phi _f',1}^\frac{1}{\alpha +1}n^{\frac{\alpha }{\alpha +1}}+\sum \limits _{j=2}^{M} \frac{a_{\rho ,j}}{n^{\nu _{g,j}}} \right) \exp \left( \sum \limits _{j=M+1}^{N_\rho } \frac{a_{\rho ,j}}{n^{\nu _{g,j}}} + O_R\left( n^{-\frac{R}{\alpha +1}}\right) \right) \end{aligned}$$

with $-\frac{\alpha }{\alpha +1}<\nu _{g,2}<\dots<\nu _{g,M}\le 0<\nu _{g,M+1}<\dots <\nu _{g,N_\rho }$. By Lemma 3.2 we obtain $a_{-\Phi _f',1}^\frac{1}{\alpha +1}=(\omega _\alpha \Gamma (\alpha +1)\zeta (\alpha +1))^\frac{1}{\alpha +1}$.

Note that the exponents $0<\nu _{g,M+1}<\dots <\nu _{g,N_\rho }$ run through

$$\begin{aligned} \left( -\frac{\alpha }{\alpha +1}-\sum _{\begin{array}{c} \mu \in {\mathcal {P}}_R \end{array}} \left( \frac{\mu +1}{\alpha +1}-1\right) {\mathbb {N}}_0\right) \cap \left( 0,\frac{R}{\alpha +1}\right) . \end{aligned}$$

By Lemma 2.4, $\exp (\sum _{j=M+1}^{N_\rho }\frac{a_{\rho ,j}}{n^{\nu _{g,j}}}+O_R(n^{-\frac{R}{\alpha +1}}))$ is in ${\mathcal {K}}(\frac{R}{\alpha +1})$, with exponent set

$$\begin{aligned} \left\{ \sum _{j=1}^K b_j\theta _j : K,b_j\in {\mathbb {N}}_0,\ \theta _j\in \left( -\frac{\alpha }{\alpha +1}-\sum _{\begin{array}{c} \mu \in {\mathcal {P}}_R \end{array}} \left( \frac{\mu +1}{\alpha +1}-1\right) {\mathbb {N}}_0\right) \cap \left( 0,\frac{R}{\alpha +1}\right) \right\} . \end{aligned}$$

As $\alpha \in {\mathcal {P}}_R$, this is a subset of ${\mathcal {N}}$, so the above exponents are given by ${\mathcal {N}}$, proving the lemma. $\square$

The following corollary is very helpful to prove our main theorem.

#### Corollary 3.9

Let $f :{\mathbb {N}}\rightarrow {\mathbb {N}}_0$ satisfy the conditions of Theorem 1.4. Then we have

$$\begin{aligned} e^{n\rho _n} G_f(\rho _n)&= \frac{e^{L_f'(0)}n^{\frac{L_f(0)}{\alpha +1}}}{a_{-\Phi _f',1}^{\frac{L_f(0)}{\alpha +1}}} \exp \left( A_1 n^{\frac{\alpha }{\alpha +1}} + \sum _{j=2}^M A_j n^{\alpha _j}\right) \\&\quad \times \left( 1 + \sum _{j = 1}^N \frac{E_j}{n^{\eta _j}} + O_R\left( n^{-\frac{R}{\alpha +1}}\right) \right) , \end{aligned}$$

with $A_1$ defined in (1.12), $\frac{\alpha }{\alpha +1}>\alpha _2>\dots >\alpha _M\ge 0$ running through ${\mathcal {L}}$, and $\eta _j$ through ${\mathcal {M}}+{\mathcal {N}}$.

## Proof of Theorem 1.4

### The general case

The following lemma follows by a straightforward calculation, using (3.1) and Lemmas 3.1, 3.5, and 3.6.

#### Lemma 4.1

Let $f:{\mathbb {N}}\rightarrow {\mathbb {N}}_0$ satisfy the conditions of Theorem 1.4. Then we have

$$\begin{aligned} p_f(n) = \frac{e^{n\rho _n}G_f(\rho _n)}{\sqrt{2\pi }}\left( \sum \limits _{j=1}^M \frac{d_j }{n^{\nu _j}} + O_{L,R}\left( n^{-\min \left\{ \frac{L+1}{\alpha +1}, \frac{R+\alpha }{\alpha +1}+\frac{\alpha +2}{2(\alpha +1)}\right\} }\right) \right) \end{aligned}$$

for some $M\in {\mathbb {N}}$, $d_1=\frac{1}{\sqrt{\alpha +1}}(\omega _\alpha \Gamma (\alpha +1)\zeta (\alpha +1))^\frac{1}{2(\alpha +1)}$, and the $\nu _j$ run through

$$\begin{aligned} \frac{\alpha +2}{2(\alpha +1)} + \frac{\alpha }{\alpha +1}{\mathbb {N}}_0 + \left( -\sum _{\mu \in {\mathcal {P}}_R} \left( \frac{\mu +1}{\alpha +1}-1\right) {\mathbb {N}}_0\right) \cap \left[ 0,\frac{R+\alpha }{\alpha +1}\right) . \end{aligned}$$

In particular, we have $\nu _1 =\tfrac{\alpha +2}{2(\alpha +1)}$.

We prove the following lemma.

#### Lemma 4.2

Assume that *f* satisfies the conditions of Theorem 1.4 and that $L_f$ has only one positive pole $\alpha$. Then we have

$$\begin{aligned} n \varrho _n + \Phi _f(\varrho _n)&= \left( \omega _{\alpha } \Gamma (\alpha +1)\zeta (\alpha +1)\right) ^{\frac{1}{\alpha +1}} \left( 1 + \tfrac{1}{\alpha }\right) n^{\frac{\alpha }{\alpha +1}} \\&\quad - L_f(0) \log (\varrho _n) + L'_f(0) + o(1). \end{aligned}$$

#### Proof

By Lemma 3.2, we have

4.1 $$\begin{aligned} \Phi _f(\varrho _n) = \frac{\omega _{\alpha } \Gamma (\alpha )\zeta (\alpha +1)}{\varrho _n^\alpha } - L_f(0)\log (\varrho _n) + L'_f(0) + O\left( \varrho _n^{R_0}\right) , \end{aligned}$$

where

4.2 $$\begin{aligned} R_0 := {\left\{ \begin{array}{ll} \underset{\nu \in {\mathcal {P}}_R\cap (-R,0)}{-\max \nu } &{} \text {if }{\mathcal {P}}_R\cap (-R,0)\ne \emptyset ,\\ R &{} \text {otherwise}. \end{array}\right. } \end{aligned}$$

To show the lemma, we need an expansion for $\rho _n$. We have, by (3.3) and again by Lemma 3.2,

$$\begin{aligned} -\Phi '_f(\varrho _n) = \frac{\omega _{\alpha } \Gamma (\alpha +1) \zeta (\alpha +1)}{\varrho _n^{\alpha +1}} + \frac{L_f(0)}{\varrho _n} + O\left( \varrho _n^{{R_0}-1}\right) . \end{aligned}$$

By Corollary 3.4, we have an expansion for $\rho _n$ with an error *o*(1). We iteratively find the first terms. By Corollary 3.4 we have $\rho _n\sim a_{-\Phi _f',1}^\frac{1}{\alpha +1}n^{-\frac{1}{\alpha +1}}$, as $n\rightarrow \infty$. We next determine the second order term in $\rho _n=\frac{a_{-\Phi _f',1}^\frac{1}{\alpha +1}}{n^\frac{1}{\alpha +1}}+\frac{K_2}{n^{\kappa _2}}+o(n^{-\kappa _2})$ for some $\kappa _2<\frac{1}{\alpha +1}$ and $K_2\in {\mathbb {C}}$. We choose $\kappa$ in

$$\begin{aligned}&n \left( 1 + \frac{K_2}{a_{-\Phi _f',1}^{\frac{1}{\alpha +1}}n^{\kappa _2 - \frac{1}{\alpha +1}}}\right) ^{-\alpha -1} + \frac{L_f(0)}{a_{-\Phi _f',1}^{\frac{1}{\alpha +1}}} n^{\frac{1}{\alpha +1}} \left( 1 + \frac{K_2}{a_{-\Phi _f',1}^{\frac{1}{\alpha +1}} n^{\kappa _2 - \frac{1}{\alpha +1}}}\right) ^{-1} \\&\quad = n + O(n^\kappa ) \end{aligned}$$

as small as possible. One finds that

$$\begin{aligned} \frac{(\alpha +1)K_2}{a_{-\Phi _f',1}^{\frac{1}{\alpha +1}}} n^{1-\kappa _2+\frac{1}{\alpha +1}} = \frac{L_f(0)}{a_{-\Phi _f',1}^{\frac{1}{\alpha +1}}}n^{\frac{1}{\alpha +1}}, \end{aligned}$$

and hence

4.3 $$\begin{aligned} \varrho _n = \frac{a_{-\Phi _f',1}^{\frac{1}{\alpha +1}}}{n^{\frac{1}{\alpha +1}}} + \frac{L_f(0)}{(\alpha +1)n} + o\left( \frac{1}{n}\right) . \end{aligned}$$

Plugging (4.3) into $\Phi _f$ leads, by (4.1), to

$$\begin{aligned}{} & {} \Phi _f\left( \frac{a_{-\Phi _f',1}^\frac{1}{\alpha +1}}{n^\frac{1}{\alpha +1}}+\frac{L_f(0)}{(\alpha +1)n}+o\left( \frac{1}{n}\right) \right) \\{} & {} \quad = \frac{a_{-\Phi _f',1}^\frac{1}{\alpha +1}}{\alpha }n^\frac{\alpha }{\alpha +1} - \frac{L_f(0)}{\alpha +1} - L_f(0)\log (\rho _n) + L_f'(0) + o(1). \end{aligned}$$

As a result, using (4.3), we conclude the claim. $\square$

We are now ready to prove Theorem 1.4.

#### Proof of Theorem 1.4

Corollaries 3.4 and 3.9 with Lemmas 4.1 and 4.2 give the asymptotic for $p_f(n)$. We use Lemma 3.7 to calculate the exponents and (1.12) as well as (1.13) for the constants. Throughout we use Lemma 2.3 2 to deal with the expansions of products of functions. $\square$

### The case of two positive poles of $L_f$

If $\alpha >0$ is the only positive pole of $L_f$, then we can calculate the single term in the exponential in the asymptotic of $p_f(n)$ explicitly, by Theorem 1.4. In this subsection, we assume that $L_f$ has exactly two positive simple poles, $\alpha$ and $\beta$. In this case, Lemma 3.2 with $k=1$ gives

$$\begin{aligned} -\Phi '_f(z) = \frac{c_1}{z^{\alpha +1}} + \frac{c_2}{z^{\beta +1}} + \frac{c_3}{z} + O_R\left( |z|^{R_0-1}\right) \end{aligned}$$

with $R_0$ from (4.2). Above we set $c_j:=a_{-\Phi _f',j}$ for $1\le j\le 3$, i.e., by Lemma 3.2

4.4 $$\begin{aligned} c_1 = \omega _{\alpha } \Gamma (\alpha +1)\zeta (\alpha +1), \quad c_2 = \omega _{\beta } \Gamma (\beta +1) \zeta (\beta +1), \quad c_3 = L_f(0). \end{aligned}$$

In the next lemma, we approximate the saddle point in this special situation.

#### Lemma 4.3

Let *f* satisfy the conditions of Theorem 1.4. Additionally assume that $L_f$ has exactly two positive poles $\alpha$ and $\beta$ that satisfy $\frac{\ell +1}{\ell }\beta <\alpha \le \frac{\ell }{\ell -1}\beta$ for some $\ell \in {\mathbb {N}}$, where we treat the case $\ell =1$ simply as $2\beta <\alpha$. Then there exists $0<r\le \frac{R}{\alpha +1}$ such that

4.5 $$\begin{aligned} \rho _n = \sum _{j=1}^{\ell +1} \frac{K_j}{n^{(j-1)\left( 1-\frac{\beta +1}{\alpha +1}\right) +\frac{1}{\alpha +1}}} + \frac{c_3}{(\alpha +1)n} + O_R\left( n^{-r-1}\right) \end{aligned}$$

for some constants $K_j$ independent of *n* and $c_3$ as in (4.4). In particular, we have

$$\begin{aligned} K_1&= c_1^\frac{1}{\alpha +1}, \hspace{3mm} K_2 = \frac{c_2}{(\alpha +1)c_1^\frac{\beta }{\alpha +1}}, \hspace{3mm} K_3 = \frac{c_2^2(\alpha -2\beta )}{2(\alpha +1)^2c_1^\frac{2\beta +1}{\alpha +1}}, \\ K_4&= \frac{c_2^3\left( 2\alpha ^2-9\alpha \beta -2\alpha +9\beta ^2+3\beta \right) }{6(\alpha +1)^3c_1^\frac{3\beta +2}{\alpha +1}}, \\ K_5&= \frac{c_2^4(6 \alpha ^3-44 \alpha ^2 \beta -15 \alpha ^2+96 \alpha \beta ^2+56 \alpha \beta +6 \alpha -64 \beta ^3-48 \beta ^2-8 \beta )}{24(\alpha +1)^4 c_1^{\frac{4\beta + 3}{\alpha + 1}}}. \end{aligned}$$

#### Proof

By Corollary 3.4, the exponents of $\rho _n$ that are at most 1 are given by combinations

$$\begin{aligned} \frac{1}{\alpha +1} + (j-1)\left( 1-\frac{\beta +1}{\alpha +1}\right) + m\left( 1-\frac{1}{\alpha +1}\right) \le 1, \end{aligned}$$

with $j\in {\mathbb {N}}$ and $m\in {\mathbb {N}}_0$. A straightforward calculation shows that $\frac{\ell +1}{\ell }\beta <\alpha \le \frac{\ell }{\ell -1}\beta$ if and only if

$$\begin{aligned} 0 < \frac{1}{\alpha +1} + (j-1)\left( 1-\frac{\beta +1}{\alpha +1}\right) \le 1 \end{aligned}$$

for all $1\le j\le \ell +1$ but not for $j>\ell +1$. Together with the error term induced by Corollary 3.4, (4.5) follows. Assuming $\ell \ge 5$, $K_1$–$K_5$ and the term $\frac{c_3}{(\alpha +1)n}$ can be determined iteratively. $\square$

We are now ready to prove asymptotic formulas if $L_f$ has exactly two positive poles.

#### Theorem 4.4

Assume that $f:{\mathbb {N}}\rightarrow {\mathbb {N}}_0$ satisfies the conditions of Theorem 1.4 and that $L_f$ has exactly two positive poles $\alpha >\beta$, such that $\frac{\ell +1}{\ell }\beta <\alpha \le \frac{\ell }{\ell -1}\beta$ for some $\ell \in {\mathbb {N}}$. Then we have

$$\begin{aligned} p_f(n){} & {} = \frac{C}{n^b}\exp \left( A_1n^\frac{\alpha }{\alpha +1}+A_2n^\frac{\beta }{\alpha +1}+\sum _{k=3}^{\ell +1} A_kn^{\frac{(k-1)\beta }{\alpha +1}+\frac{k-2}{\alpha +1}+2-k}\right) \\{} & {} \quad \times \left( 1+\sum _{j=2}^{M_1} \frac{B_j}{n^{\nu _j}} + O_{L,R}\left( n^{-\min \left\{ \frac{2L-\alpha }{2(\alpha +1)},\frac{R}{\alpha +1}\right\} }\right) \right) ,\qquad (n\rightarrow \infty ), \end{aligned}$$

with

4.6 $$\begin{aligned}{} & {} A_1 := (\omega _{\alpha }\Gamma (\alpha +1)\zeta (\alpha +1))^\frac{1}{\alpha +1}\left( 1+\frac{1}{\alpha }\right) ,\nonumber \\{} & {} A_2 := \frac{\omega _{\beta }\Gamma (\beta )\zeta (\beta +1)}{(\omega _\alpha \Gamma (\alpha +1)\zeta (\alpha +1))^\frac{\beta }{\alpha +1}}, \end{aligned}$$

and for all $k \ge 3$

$$\begin{aligned} A_k := K_k + \frac{c_1^\frac{1}{\alpha +1}}{\alpha }\sum _{m=1}^\ell \left( {\begin{array}{c}-\alpha \\ m\end{array}}\right) \sum _{\begin{array}{c} 0\le j_1,\dots ,j_\ell \le m\\ j_1+\cdots +j_\ell =m\\ j_1+2j_2+\cdots +\ell j_\ell =k-1 \end{array}} \left( {\begin{array}{c}m\\ j_1,j_2,\dots ,j_\ell \end{array}}\right) \frac{K_2^{j_1}\ldots K_{\ell +1}^{j_\ell }}{c_1^\frac{m}{a+1}}\\ + \frac{c_2}{\beta c_1^\frac{\beta }{a+1}}\sum _{m=1}^\ell \left( {\begin{array}{c}-\beta \\ m\end{array}}\right) \sum _{\begin{array}{c} 0\le j_1,\dots ,j_\ell \le m\\ j_1+\cdots +j_\ell =m\\ j_1+2j_2+\cdots +\ell j_\ell =k-2 \end{array}} \left( {\begin{array}{c}m\\ j_1,j_2,\dots ,j_\ell \end{array}}\right) \frac{K_2^{j_1}\ldots K_{\ell +1}^{j_\ell }}{c_1^\frac{m}{a+1}}. \end{aligned}$$

Here, *C* and *b* are defined in (1.12) and (1.13), the $\nu _j$ run through ${\mathcal {M}}+{\mathcal {N}}$, the $K_j$ are given in Lemma 4.3, and $c_1$, $c_2$, and $c_3$ run through (4.4).

#### Proof

Assume that $g:{\mathbb {N}}\rightarrow {\mathbb {C}}$ has an asymptotic expansion as $n\rightarrow \infty$ and denote by $[g(n)]_*$ the part with nonnegative exponents. With Lemmas 3.2 and 4.1 we obtain, using that $L_f$ has exactly two positive poles in $\alpha$ and $\beta$,

$$\begin{aligned} p_f(n)&= \frac{C}{n^b} \exp \left( \left[ n\varrho _n + \frac{c_1}{\alpha \varrho _n^\alpha } + \frac{c_2}{\beta \varrho _n^\beta }\right] _* \right) \\&\quad \times \left( 1+\sum \limits _{j=2}^{M_1} \frac{a_j }{n^{\delta _j}} + O_{L,R}\left( n^{-\min \left\{ \frac{2L-\alpha }{2(\alpha +1)}, \frac{R}{\alpha +1}\right\} }\right) \right) \end{aligned}$$

with the $\delta _j$ running through ${\mathcal {M}}$. With the Binomial Theorem and Lemma 4.3, we find

4.7 $$\begin{aligned} \hspace{-12pt}\frac{c_1}{\alpha \rho _n^\alpha } {=} \frac{c_1^\frac{1}{\alpha +1}}{\alpha }n^\frac{\alpha }{\alpha +1}\left( 1 {+} \hspace{-.1cm}\sum _{m\ge 1}\hspace{-.1cm} \left( {\begin{array}{c}-\alpha \\ m\end{array}}\right) \left( \sum _{j=2}^{\ell +1} \frac{K_jc_1^{-\frac{1}{\alpha {+}1}}}{n^{(j-1)\left( 1{-}\frac{\beta +1}{\alpha +1}\right) }} {+} \frac{c_3c_1^{-\frac{1}{\alpha +1}}}{(\alpha {+}1)n^\frac{\alpha }{\alpha +1}} {+} o\left( n^{-\frac{\alpha }{\alpha +1}}\right) \right) ^m\hspace{.1cm}\right) . \end{aligned}$$

By definition, $[\frac{c_1}{\alpha \rho _n^\alpha }]_*$ is the part of the expansion of $\frac{c_1}{\alpha \rho _n^\alpha }$ involving nonnegative powers of *n*, i.e., for $m\ge 2$ in the sum on the right of (4.7) we can ignore the term

$$\begin{aligned} \frac{c_3}{(\alpha +1)c_1^\frac{1}{\alpha +1}n^\frac{\alpha }{\alpha +1}} + o\left( n^{-\frac{\alpha }{\alpha +1}}\right) . \end{aligned}$$

Applying the Multinomial Theorem to (4.7) gives

4.8 $$\begin{aligned} \frac{c_1}{\alpha \rho _n^\alpha }{} & {} = \frac{c_1^\frac{1}{\alpha +1}}{\alpha }n^\frac{\alpha }{\alpha +1} - \frac{c_3}{\alpha +1} + \frac{c_1^\frac{1}{\alpha +1}}{\alpha }\sum _{m=1}^\ell \left( {\begin{array}{c}-\alpha \\ m\end{array}}\right) \nonumber \\{} & {} \quad \times \sum _{\begin{array}{c} 0\le j_1,j_2,\dots ,j_\ell \le m\\ j_1+\dots +j_\ell =m \end{array}} \left( {\begin{array}{c}m\\ j_1,j_2,\dots ,j_\ell \end{array}}\right) \frac{K_2^{j_1}\ldots K_{\ell +1}^{j_\ell }}{c_1^\frac{m}{a+1}}\nonumber \\{} & {} \quad \times n^{\frac{(j_1+2j_2+\dots +\ell j_\ell )\beta }{\alpha +1}+\frac{j_1+2j_2+\dots +\ell j_\ell -1}{\alpha +1}-(j_1+2j_2+\dots +\ell j_\ell -1)} + o(1). \end{aligned}$$

Similarly, we have

4.9 $$\begin{aligned} \frac{c_2}{\beta \rho _n^\beta }{} & {} = \frac{c_2}{\beta c_1^\frac{\beta }{a+1}}n^\frac{\beta }{\alpha +1} + \frac{c_2}{\beta c_1^\frac{\beta }{a+1}}\sum _{m=1}^\ell \left( {\begin{array}{c}-\beta \\ m\end{array}}\right) \nonumber \\{} & {} \quad \times \sum _{\begin{array}{c} 0\le j_1,j_2,\dots ,j_\ell \le m\\ j_1+\dots +j_\ell =m \end{array}} \left( {\begin{array}{c}m\\ j_1,j_2,\dots ,j_\ell \end{array}}\right) \frac{K_2^{j_1}\cdots K_{\ell +1}^{j_\ell }}{c_1^\frac{m}{a+1}}\nonumber \\{} & {} \quad \times n^{\frac{(j_1+2j_2+\dots +\ell j_\ell +1)\beta }{\alpha +1}+\frac{j_1+2j_2+\dots +\ell j_\ell }{\alpha +1}-(j_1+2j_2+\dots +\ell j_\ell )} + o(1). \end{aligned}$$

Finally, we obtain, with Lemma 4.3,

4.10 $$\begin{aligned}{}[n\rho _n]_*= K_1n^\frac{\alpha }{\alpha +1} + \sum _{m=1}^\ell K_{m+1}n^{\frac{m\beta }{\alpha +1}+\frac{m-1}{\alpha +1}-(m-1)} +\frac{c_3}{\alpha +1}. \end{aligned}$$

Combining (4.8), (4.9), and (4.10), we find that

$$\begin{aligned} \left[ n\rho _n+\frac{c_1}{\alpha \rho _n^\alpha }+\frac{c_2}{\beta \rho _n^\beta }\right] _* = \left( 1+\frac{1}{\alpha }\right) c_1^\frac{1}{\alpha +1}n^\frac{\alpha }{\alpha +1} + \frac{c_2}{\beta c_1^\frac{\beta }{\alpha +1}}n^\frac{\beta }{\alpha +1} + \sum _{k=2}^\ell A_{k+1}n^{\frac{k\beta }{\alpha +1}+\frac{k-1}{\alpha +1}-(k-1)}, \end{aligned}$$

where

$$\begin{aligned} A_k{} & {} = K_k + \frac{c_1^\frac{1}{\alpha +1}}{\alpha }\sum _{m=1}^\ell \left( {\begin{array}{c}-\alpha \\ m\end{array}}\right) \sum _{\begin{array}{c} 0\le j_1,j_2,\dots ,j_\ell \le m\\ j_1+\dots +j_\ell =m\\ j_1+2j_2+\dots +\ell j_\ell =k-1 \end{array}} \left( {\begin{array}{c}m\\ j_1,j_2,\dots ,j_\ell \end{array}}\right) \frac{K_2^{j_1}\ldots K_{\ell +1}^{j_\ell }}{c_1^\frac{m}{a+1}}\\{} & {} \quad + \frac{c_2}{\beta c_1^\frac{\beta }{a+1}}\sum _{m=1}^\ell \left( {\begin{array}{c}-\beta \\ m\end{array}}\right) \sum _{\begin{array}{c} 0\le j_1,j_2,\dots ,j_\ell \le m\\ j_1+\dots +j_\ell =m\\ j_1+2j_2+\dots +\ell j_\ell =k-2 \end{array}} \left( {\begin{array}{c}m\\ j_1,j_2,\dots ,j_\ell \end{array}}\right) \frac{K_2^{j_1}\ldots K_{\ell +1}^{j_\ell }}{c_1^\frac{m}{a+1}}. \end{aligned}$$

Note that we have by definition of $c_1$, $c_2$ (see (4.4)), $K_1$, and $K_2$ (see Lemma 4.3),

$$\begin{aligned} A_1&= \left( 1+\frac{1}{\alpha }\right) c_1^\frac{1}{\alpha +1} = \left( 1+\frac{1}{\alpha }\right) (\omega _\alpha \Gamma (\alpha +1)\zeta (\alpha +1))^\frac{1}{\alpha +1},\\ A_2&= \frac{c_2}{\beta c_1^\frac{\beta }{a+1}} = \frac{\omega _\beta \Gamma (\beta )\zeta (\beta +1)}{(\omega _\alpha \Gamma (\alpha +1)\zeta (\alpha +1))^\frac{\beta }{\alpha +1}}, \end{aligned}$$

which gives (4.6). Hence we indeed obtain, as $n\rightarrow \infty$, for suitable $M_1\in {\mathbb {N}}$

$$\begin{aligned} p_f(n)&= \frac{C}{n^b}\exp \left( A_1 n^{\frac{\alpha }{\alpha +1}} + A_2 n^{\frac{\beta }{\alpha +1}} + \sum _{k=3}^{\ell +1} A_k n^{\frac{(k-1)\beta }{\alpha +1}+\frac{k-2}{\alpha +1}-(k-2)} \right) \\&\quad \times \left( 1+\sum \limits _{j=2}^{M_1} \frac{B_j}{n^{\nu _j}} + O_{L,R}\left( n^{-\min \left\{ \frac{2L-\alpha }{2(\alpha +1)}, \frac{R}{\alpha +1}\right\} }\right) \right) , \end{aligned}$$

where the $\nu _j$ run, as in Theorem 1.4, through ${\mathcal {M}}+{\mathcal {N}}$. This proves the theorem. $\square$

## Proofs of Theorems 1.1, 1.2, and 1.3

We require the zeta function associated to a polynomial *P*,

$$\begin{aligned} Z_P(s) := \sum _{n\ge 1} \frac{1}{P(n)^s} \end{aligned}$$

with $P(n)>0$ for $n\in {\mathbb {N}}$. In particular, we consider $P=P_k$ (see (1.4)). The following lemma ensures that all the $P_k$ satisfy (P1) with *L* arbitrary large.

### Lemma 5.1

Let $k \ge 3$ be an integer and let

$$\begin{aligned} \Lambda ^{[k]} := \left\{ P_k(n) : n \in {\mathbb {N}}\right\} . \end{aligned}$$

For every prime *p*, we have $|\Lambda ^{[k]}\setminus (\Lambda ^{[k]}\cap p{\mathbb {N}})|=\infty$.

We next show that (P2) and (P3) hold.

### Proposition 5.2

Let $$k\in {\mathbb {N}}$$ with $$k\ge 3$$.

1. The function $Z_{P_k}$ has a meromorphic continuation to ${\mathbb {C}}$ with at most simple poles in $\frac{1}{2}-{\mathbb {N}}_0$. The positive pole lies in $s=\frac{1}{2}$.
2. We have $Z_{P_k}(s) \ll Q_k(|\textrm{Im}(s)|)$ as $|\textrm{Im}(s)|\rightarrow \infty$ for some polynomial $Q_k$.

### Proof

(1) The meromorphic continuation of $Z_{P_k}$ to ${\mathbb {C}}$ follows by [29, Theorem B]. By [29, Theorem A (ii)] the only possible poles (of order at most one) are located at $\frac{1}{2}-\frac{1}{2}{\mathbb {N}}_0$. Holomorphicity in $-{\mathbb {N}}_0$ is a direct consequence of [29, Theorem C]. Finally, note that $P_k(n) \ll _k n^2$. Thus, as $x\rightarrow \infty$,

$$\begin{aligned} \sum _{1\le n \le x} \frac{1}{P_k(n)^{\frac{1}{2}}} \gg _k \sum _{1\le n \le x} \frac{1}{n}. \end{aligned}$$

This proves the existence of a pole in $s = \frac{1}{2}$, completing the proof.

(2) This result follows directly by [29, Proposition 1 (iii)]. $\square$

To apply Theorem 1.4, it remains to compute $Z_{P_k}(0)$ and $Z_{P_k}'(0)$, as well as ${\text {Res}}_{s=\frac{1}{2}} Z_{P_k}(s)$.

### Proposition 5.3

Let $$k\in {\mathbb {N}}$$ with $$k\ge 3$$.

1. We have $Z_{P_k}(0)= \frac{1}{2-k}$ and
  $$\begin{aligned} Z_{P_k}'(0) = \frac{\log \left( \frac{k-2}{2}\right) }{k-2} + \log \left( \Gamma \left( \frac{2}{k-2}\right) \right) - \log (2\pi ). \end{aligned}$$
2. We have $${\text {Res}}_{s=\frac{1}{2}}Z_{P_k}(s)=\sqrt{\frac{1}{2(k-2)}}$$.

### Proof

(1) Since the roots of $P_k$ are not in ${\mathbb {R}}_{\ge 1}$, we may use [29, Theorem D] to obtain that $Z_{P_k}(0)=\frac{1}{2-k}$. For the derivative, one applies [29, Theorem E] yielding

$$\begin{aligned} Z_{P_k}'(0) = \frac{\log \left( \frac{k-2}{2}\right) }{k-2} + \log \left( \Gamma \left( \frac{2}{k-2}\right) \right) - \log (2\pi ). \end{aligned}$$

(2) Since $Z_{P_k}(s)=(\frac{2}{k-2})^s\sum \limits _{n\ge 1}(n-\frac{k-4}{k-2})^{-s}n^{-s}$, the result follows as the sum has residue $\frac{1}{2}$ at $s=\frac{1}{2}$ by equation (16) of [29]. $\square$

The previous three lemmas are used to prove Theorem 1.1.

### Proof of Theorem 1.1

We may apply Theorem 1.4 as Lemma 5.1 and Proposition 5.2 ensure that conditions (P1)–(P3) are satisfied. Hence, one obtains an asymptotic formula for $p_k(n)$. The constants occurring in Theorem 1.4 are computed using (1.12), (1.13), and Proposition 5.3. That the exponential consists only of the term $A_1 n^\frac{1}{3}$ follows by Theorem 1.4, since $Z_{P_k}(s)$ has exactly one positive pole, lying in $s=\frac{1}{2}$. Note that we are allowed to choose *L* and *R* arbitrarily large due to Lemma 5.1 and Proposition 5.2 (1). $\square$

We consider some special cases of Theorem 1.1.

### Corollary 5.4

For triangular numbers, squares, and pentagonal numbers, respectively, we have

$$\begin{aligned} p_3(n)&\sim \frac{\zeta \left( \frac{3}{2}\right) }{2^\frac{7}{2}\sqrt{3}\pi n^\frac{3}{2}}\exp \left( \frac{3}{2}\pi ^\frac{1}{3}\zeta \left( \frac{3}{2}\right) ^\frac{2}{3}n^\frac{1}{3}\right) ,\\ p_4(n)&\sim \frac{\zeta \left( \frac{3}{2}\right) ^\frac{2}{3}}{2^\frac{7}{3}\sqrt{3}\pi ^\frac{7}{6}n^\frac{7}{6}} \exp \left( \frac{3}{2^\frac{4}{3}}\pi ^\frac{1}{3}\zeta \left( \frac{3}{2}\right) ^\frac{2}{3}n^\frac{1}{3}\right) ,\\ p_5(n)&\sim \frac{\Gamma \left( \frac{2}{3}\right) \zeta \left( \frac{3}{2}\right) ^\frac{5}{9}}{2^\frac{13}{6}3^\frac{4}{9}\pi ^\frac{11}{9}n^\frac{19}{18}} \exp \left( \frac{3^\frac{2}{3}}{2}\pi ^\frac{1}{3}\zeta \left( \frac{3}{2}\right) ^\frac{2}{3}n^\frac{1}{3}\right) . \end{aligned}$$

The next lemma shows that $\prod _{j,k\ge 1}({1-q^\frac{jk(j+k)(j+2k)}{6}})^{-1}$ satisfies (P1) for *L* arbitrarily large.

### Lemma 5.5

Let $f :{\mathbb {N}}\rightarrow {\mathbb {N}}_0$ be defined by

$$\begin{aligned} f(n) := \left| \left\{ (j,k)\in {\mathbb {N}}^2 : \frac{jk(j+k)(j+2k)}{6}=n\right\} \right| . \end{aligned}$$

Then, for all primes *p*, we have $|\Lambda \setminus (\Lambda \cap p{\mathbb {N}})| = \infty$.

For investigating the function $\zeta _{\mathfrak {so}(5)}$, we need the *Mordell–Tornheim zeta function*, defined by

$$\begin{aligned} \zeta _{{\text {MT}},2}(s_1,s_2,s_3) := \sum _{m,n\ge 1} m^{-s_1} n^{-s_2} (m+n)^{-s_3}. \end{aligned}$$

By [27], for $\textrm{Re}(s)>1$ and some $-\textrm{Re}(s)<c<0$ we get a relation between $\zeta _{{\text {MT}},2}$ and $\zeta _{\mathfrak {so}(5)}$ via

5.1 $$\begin{aligned} \zeta _{\mathfrak {so}(5)}(s) = \frac{6^s}{2\pi i \Gamma (s)} \int _{c - i\infty }^{c + i\infty } \Gamma (s+z)\Gamma (-z) \zeta _{{\text {MT}},2}(s,s-z,2s+z)dz. \end{aligned}$$

We have the following theorem.

### Theorem 5.6

[26, Theorem 1] The function $\zeta _{{\text {MT}}, 2}$ has a meromorphic continuation to ${\mathbb {C}}^3$ and its singularities satisfy $s_1 + s_3 = 1-\ell , s_2 + s_3 = 1-\ell , s_1 + s_2 + s_3 = 2,$ with $\ell \in {\mathbb {N}}_0$.

Fix $M\in {\mathbb {N}}_0$ and $0<\varepsilon <1$. Let $\textrm{Re}(s_1),\textrm{Re}(s_3)>1$, $\textrm{Re}(s_2)>0$, and $s_2\notin {\mathbb {N}}$. Then, for $\textrm{Re}(s_2)<M+1-\varepsilon$, we have (see equation (5.3) in [26])

5.2 $$\begin{aligned} \zeta _{{\text {MT}},2}(s_1, s_2, s_3)&= \frac{\Gamma (s_2+s_3-1)\Gamma (1-s_2)}{\Gamma (s_3)} \zeta (s_1 + s_2 + s_3 - 1) \nonumber \\&\hspace{0.35cm} + \sum _{m=0}^{M-1} {-s_3 \atopwithdelims ()m} \zeta (s_1+s_3+m)\zeta (s_2 - m) \nonumber \\&\quad + \frac{1}{2\pi i} \int _{M-\varepsilon - i\infty }^{M-\varepsilon {+} i\infty } \frac{\Gamma (s_3 {+} w)\Gamma (-w)}{\Gamma (s_3)} \zeta (s_1 {+} s_3 {+} w)\zeta (s_2 {-} w)dw. \end{aligned}$$

The first two summands on the right-hand side of (5.2) extend meromorphically to ${\mathbb {C}}^3$, so to show that (5.1) extends meromorphically, we consider (5.2). Note that $\textrm{Re}(w)=M-\varepsilon$. To avoid poles on the line of integration, we assume that

5.3 $$\begin{aligned} \textrm{Re}(s_3 + w)> 0&\Leftrightarrow \textrm{Re}(s_3) > \varepsilon -M, \end{aligned}$$

5.4 $$\begin{aligned} \textrm{Re}(s_1+s_3+w)> 1&\Leftrightarrow \textrm{Re}(s_1) + \textrm{Re}(s_3) > 1 - M + \varepsilon , \end{aligned}$$

5.5 $$\begin{aligned} \textrm{Re}(s_2 - w)< 1&\Leftrightarrow \textrm{Re}(s_2) < 1 + M - \varepsilon . \end{aligned}$$

Note that the final condition is already assumed above.

By Propostition 2.6 (2), the integral converges compactly and the integrands are locally holomorphic. Thus, the integral is a holomorphic function in the region defined by (5.3), (5.4), and (5.5). Recalling (5.1), we are interested in $\zeta _{{\text {MT}},2}(s,s-z,2s+z)$. By Theorem 5.6, this function is meromorphic in ${\mathbb {C}}^2$ and holomorphic outside the hyperplanes defined by $3s+z=1-\ell$, $3s=1-\ell$, and $4s=2$, where $\ell \in {\mathbb {N}}_0$. With (5.2), we obtain

5.6 $$\begin{aligned} \zeta _{{\text {MT}},2}(s,s-z,2s+z){} & {} = \frac{\Gamma (3s-1)\Gamma (z+1-s)}{\Gamma (2s+z)}\zeta (4s-1)\nonumber \\{} & {} \quad + \sum _{m=0}^{M-1} \left( {\begin{array}{c}-2s{-}z\\ m\end{array}}\right) \zeta (3s{+}z{+}m)\zeta (s{-}z{-}m) {+} I_M(s;z), \end{aligned}$$

where $$s\in {\mathbb {C}}\setminus \{\frac{1}{2},\frac{1-\ell }{3}\}$$, and

$$\begin{aligned} I_M(s; z) := \frac{1}{2\pi i} \int _{M-\varepsilon -i\infty }^{M-\varepsilon +i\infty } \frac{\Gamma (2s+z+w)\Gamma (-w)}{\Gamma (2s+z)} \zeta (3s+z+w)\zeta (s-z-w) dw. \end{aligned}$$

The following lemma shows that $I_M(s;z)$ is holomorphic in *z*. To state it let

$$\begin{aligned} \mu = \mu _{M,\sigma } := \max \{-1+\sigma -M+\varepsilon ,1-3\sigma -M+\varepsilon ,-2\sigma -M+\varepsilon \}. \end{aligned}$$

### Lemma 5.7

Let $s=\sigma +it\in {\mathbb {C}}$, $M\in {\mathbb {N}}_0$, and $0<\varepsilon <1$. Then $z\mapsto I_M(s;z)$ is holomorphic in $S_{\mu ,\infty }$.

### Proof

If $z\in S_{\mu ,\infty }$, then $\textrm{Re}(2s+z+w)>0$, $\textrm{Re}(3s+z+w)>1$, and $\textrm{Re}(s-z-w)<1$ for $w\in {\mathbb {C}}$ satisfying $\textrm{Re}(w)=M-\varepsilon$, so $\Gamma (2s+z+w)$, $\zeta (3s+z+w)$, and $\zeta (s-z-w)$ have no poles on the path of integration. As $0<\varepsilon <1$, we have $M-\varepsilon \notin {\mathbb {N}}_0$, so $w\mapsto \Gamma (-w)$ has no pole if $\textrm{Re}(w)=M-\varepsilon$. As a result, no pole is located on the path of integration, and by Proposition 2.6 (2) and the uniform polynomial growth of the zeta function along vertical strips we find that the integral converges uniformly on compact subsets of $S_{\mu ,\infty }$. $\square$

The next lemma shows, that $I_M$ is bounded polynomially in certain vertical strips. A proof is obtained using Propositions 2.6 (2) and 2.7 (2).

### Lemma 5.8

Let $\sigma _1 < \sigma _2$ and $\sigma _3 < \sigma _4$, such that $S_{\sigma _3, \sigma _4} \subset S_{\mu ,\infty }$ for all $s \in S_{\sigma _1, \sigma _2}$ and fix $0< \varepsilon < 1$ sufficiently small. In $S_{\sigma _1,\sigma _2} \times S_{\sigma _3,\sigma _4}$ the function $(s,z) \mapsto I_M(s;z)$ is holomorphic and satisfies $|I_M(s;z)|\le P_{\sigma _1,\sigma _2, \sigma _3,\sigma _4,M}(|\textrm{Im}(s)|,|\textrm{Im}(z)|)$ for some polynomial $P_{\sigma _1,\sigma _2, \sigma _3,\sigma _4,M}(X,Y)\in {\mathbb {R}}[X,Y]$.

Next we investigate $\zeta _{{\text {MT}},2}(s,s-z,2s+z)$ for fixed *s* more in detail.

### Lemma 5.9

Let $s\in {\mathbb {C}}\setminus \{\frac{1}{2},\frac{1}{3}-\frac{1}{3}{\mathbb {N}}_0\}$. Then $z\mapsto \zeta _{{\text {MT}},2}(s,s-z,2s+z)$ is holomorphic in the entire complex plane except for possibly simple poles in $z=1-\ell -3s$ with $\ell \in {\mathbb {N}}_0$.

### Proof

As holomorphicity is a local property, it suffices to consider arbitrary right half-planes. By Lemma 5.7, for *M* sufficiently large, $I_M$ is holomorphic in an arbitrary right half-plane. By (5.2), possible poles of $\zeta _{\text {MT},2}(s,s-z,2s+z)$ therefore lie in $z=s-\ell$ and in $z=-3s-m-\ell$, $\ell \in {\mathbb {N}}$. A direct calculation shows that the residue at $z=s-\ell$ vanishes if $\ell \le M-1$. Consequently, for a fixed pole $s-\ell$, we can choose *M* sufficiently large such that we only have to consider the of (5.2). This gives the claim. $\square$

We are now ready to prove growth properties of $\zeta _{{\text {MT}},2}$. As we need to avoid critical singular points, we focus on incomplete half-planes of the type $S_{\sigma _1, \sigma _2, \delta }$ (with $\delta >0$ arbitrarily small).

### Lemma 5.10

Let $\sigma _1<\sigma _2$, $\sigma _3<\sigma _4$ with $1-3\sigma _1<\sigma _3$ and $\delta > 0$ arbitrarily small. For $(s,z)\in S_{\sigma _1,\sigma _2,\delta }\times S_{\sigma _3,\sigma _4}$, we have, for some polynomial $P_{\sigma _1,\sigma _2,\sigma _3,\sigma _4,\delta }$ only depending on $S_{\sigma _1,\sigma _2,\delta }$ and $S_{\sigma _3,\sigma _4}$,

$$\begin{aligned} |\zeta _{{\text {MT}},2}(s,s-z,2s+z)| \le P_{\sigma _1,\sigma _2,\sigma _3,\sigma _4,\delta }(|\textrm{Im}(s)|,|\textrm{Im}(z)|). \end{aligned}$$

If $\sigma _1 < 0$, for all $s \in U$ with $U \subset S_{\sigma _1, \sigma _2}$, a sufficiently small neighborhood of 0, we have

$$\begin{aligned} \left| \frac{\zeta _{{\text {MT}},2}(s,s-z,2s+z)}{\Gamma (s)}\right| \le P_{\sigma _3, \sigma _4, U}(|\textrm{Im}(z)|), \end{aligned}$$

where the polynomial $P_{\sigma _3, \sigma _4, U}$ only depends on $\sigma _3$, $\sigma _4$, and *U*.

We need another lemma dealing with the poles of the Mordell–Tornheim zeta function.

### Lemma 5.11

Let $k\in {\mathbb {N}}_0$. Then the meromorphic function $s\mapsto \zeta _{{\text {MT}}, 2}(s,s-k,2s+k)$ is holomorphic for $s=-\ell$ with $\ell \in {\mathbb {N}}_{\ge \frac{k}{2}}$ and has possible simple poles at $s=\ell \in {\mathbb {N}}_0$ with $0\le \ell <\frac{k}{2}$. In particular, $s\mapsto {\Gamma (s+k)\zeta _{{\text {MT}},2}(s,s-k,2s+k)}{\Gamma (s)^{-1}}$ is holomorphic at $s=-\ell$ with $\ell \in {\mathbb {N}}_0$.

### Proof

Let *s* lie in a bounded neighborhood of $-\ell$. We use (5.6) with $s=k$. Analogous to the proof of Lemma 5.7, the function $s\mapsto I_M(s;k)$ is holomorphic in a neighborhood of $s=-\ell$. The analysis of the remaining terms is straightforward, and the lemma follows. $\square$

The next lemma states where the integral of (5.1) defining $\zeta _{\mathfrak {so}(5)}$ is a meromorphic function.

### Lemma 5.12

Let $\varepsilon >0$ be sufficiently small and let $K\in {\mathbb {N}}$. Then the function

5.7 $$\begin{aligned} s \mapsto \frac{1}{2\pi i \Gamma (s)} \int _{K - \varepsilon - i\infty }^{K - \varepsilon + i\infty } \Gamma (s+z)\Gamma (-z) \zeta _{{\text {MT}},2}(s, s-z, 2s+z)dz \end{aligned}$$

is meromorphic on the half plane $\{s\in {\mathbb {C}}:\textrm{Re}(s)>\frac{1-K+\varepsilon }{3}\}$ with at most simple poles in $\{\frac{1}{2},\frac{1}{3}-\frac{1}{3}{\mathbb {N}}_0\}\setminus (-{\mathbb {N}}_0)$ (with $\textrm{Re}(s)>\frac{1-K+\varepsilon }{3}$) and grows polynomially on vertical strips with finite width.

### Proof

We first show holomorphicity in $S_{\sigma _1,\sigma _2,\delta }$ with $\frac{1-K+\varepsilon }{3}<\sigma _1<\sigma _2$ and $0<\delta <1$. Since $\textrm{Re}(s)>\frac{1-K+\varepsilon }{3}>-K+\varepsilon$, there are no poles of $\Gamma (s+z)\Gamma (-z)$ on the path of integration $\textrm{Re}(z)=K-\varepsilon$. By Lemma 5.9, $z\mapsto \zeta _{{\text {MT}},2}(s,s-z,2s+z)$ has no poles for $s\in S_{\sigma _1,\sigma _2,\delta }$, as $\textrm{Re}(z+3s-1)=K-\varepsilon +3\textrm{Re}(s)-1>0$. By Proposition 2.6 (2), Lemmas 5.10, and 2.9, the integral is holomorphic away from singularities and grows polynomially on vertical strips of finite width.

We are left to show that (5.7) has at most a simple pole at $s=s_0$, where $s_0\in \{\frac{1}{2},\frac{1}{3}-\frac{1}{3}{\mathbb {N}}_0\}\setminus (-{\mathbb {N}}_0)$ with $s_0\ge \frac{1-K+\varepsilon }{3}$. Recall the representation of $\zeta _{{\text {MT}},2}$ in (5.6). By Lemma 5.8

$$\begin{aligned} \int _{K-\varepsilon -i\infty }^{K-\varepsilon +i\infty } \Gamma (s+z) \Gamma (-z) I_M(s;z) dz \end{aligned}$$

converges absolutely and uniformly on any sufficiently small compact subset *C* containing $s_0$ for *M* sufficiently large. Similarly, by Propositions 2.7 (2) and 2.6 (2),

$$\begin{aligned} \int _{K-\varepsilon -i\infty }^{K-\varepsilon +i\infty } \Gamma (s+z)\Gamma (-z) \sum _{m=0}^{M-1} \left( {\begin{array}{c}-2s-z\\ m\end{array}}\right) \zeta (3s+z+m)\zeta (s-z-m) dz \end{aligned}$$

converges absolutely and uniformly in *C*. In particular, both integrals continue holomorphically to $s_0$. As $s\mapsto \frac{1}{\Gamma (s)}$ is entire, it is sufficient to study

$$\begin{aligned} \frac{\Gamma (3s-1)\zeta (4s-1)}{\Gamma (s)} \int _{K-\varepsilon -i\infty }^{K-\varepsilon +i\infty } \frac{\Gamma (s+z)\Gamma (-z)\Gamma (1+z-s)}{\Gamma (2s+z)}dz \end{aligned}$$

around $s_0$. Again, by Proposition 2.6 (2), the integral converges absolutely and uniformly in *C*. As $\frac{\Gamma (3s-1)\zeta (4s-1)}{\Gamma (s)}$ has at most a simple pole in $s_0$ and a removable singularity if $s_0\in -{\mathbb {N}}_0$, the proof of the lemma is complete. $\square$

The following lemma is a refinement of Lemma 5.12 for the specific case that $z\in {\mathbb {Z}}$ and follows from Lemma 5.8, by using Propositions 2.6 and 2.7.

### Lemma 5.13

Let $k\in {\mathbb {N}}_0$ with $0\le k\le K-1$. Then, for all $\sigma _1<\sigma _2$, there exists a polynomial $P_{K,\sigma _1,\sigma _2}$, such that, uniformly for all $\sigma _1\le \textrm{Re}(s)\le \sigma _2$ and $|\textrm{Im}(s)|\ge 1$,

$$\begin{aligned} |\zeta _{{\text {MT}},2}(s,s-k,2s+k)| \le P_{K,\sigma _1,\sigma _2}(|\textrm{Im}(s)|). \end{aligned}$$

The following theorem shows that the function $\zeta _{\mathfrak {so}(5)}$ satisfies the conditions of Theorem 1.4 and gives the more precise statement of Theorem 1.2.

### Theorem 5.14

The function $\zeta _{\mathfrak {so}(5)}$ extends to a meromorphic function in ${\mathbb {C}}$ and is holomorphic in ${\mathbb {N}}_0$. For $K\in {\mathbb {N}}$ and $0< \varepsilon < 1$, we have, on $S_{\frac{1-K+\varepsilon }{3}, \infty }$,

5.8 $$\begin{aligned} \zeta _{\mathfrak {so}(5)}(s)&= \frac{6^s}{\Gamma (s)} \sum _{k=0}^{K-1} \frac{(-1)^k \Gamma (s+k)}{k!}\zeta _{{\text {MT}},2}(s, s-k, 2s+k)\nonumber \\&\quad + \frac{6^s}{2\pi i\Gamma (s)} \int _{K - \varepsilon - i\infty }^{K - \varepsilon + i\infty } \Gamma (s+z)\Gamma (-z) \zeta _{{\text {MT}},2}(s, s-z, 2s+z) dz. \end{aligned}$$

All poles of $\zeta _{\mathfrak {so}(5)}$ are simple and contained in $\{\frac{1}{2},\frac{1}{3},-\frac{1}{3},-\frac{2}{3},\dots \}$. Furthermore, for all $\sigma _0\le \sigma \le \sigma _1$ as $|\textrm{Im}(s)|\rightarrow \infty$, for some polynomial depending only on $\sigma _0$ and $\sigma _1$,

$$\begin{aligned} |\zeta _{\mathfrak {so}(5)}(s)| \le P_{\sigma _0,\sigma _1}(|\textrm{Im}(s)|). \end{aligned}$$

### Proof

Assume $\textrm{Re}(s) > 1$. By Lemma 5.9, the only poles of the integrand in (5.1) in $S_{-\textrm{Re}(s),\infty }$ lie at $z\in {\mathbb {N}}_0$. By shifting the path to the right of $\textrm{Re}(z)=M-\varepsilon$, we find, with Lemma 5.10 and the Residue Theorem, that (5.8) holds on $S_{1,\infty }$. By Lemma 5.12 the right-hand side is a meromorphic function on $S_{\frac{1-K+\varepsilon }{3},\infty }$. By Theorem 5.6, the functions $s\mapsto \zeta _{{\text {MT}},2}(s,s-k,2s+k)$ only have possible (simple) poles for $s_1+s_3=3s+k=1-\ell$, $s_2+s_3=3s =1-\ell$, $s_1+s_2+s_3=4s=2$, with $\ell \in {\mathbb {N}}_0$, i.e., for $s\in \{\frac{1}{2},\frac{1}{3},0,-\frac{1}{3},-\frac{2}{3},-1,\dots \}$. However, by Lemma 5.11 the sum in (5.8) continues holomorphically to $-{\mathbb {N}}_0$, so the sum only contributes possible poles $s\in {\mathcal {S}}:=\{\frac{1}{2},\frac{1}{3},-\frac{1}{3},-\frac{2}{3},-\frac{4}{3},\dots \}$. Note that this argument does not depend on the choice of *K*. On the other hand, if we choose *K* sufficiently large, then the integral in (5.8) is a holomorphic function around $s=-m$ for fixed but arbitrary $m\in {\mathbb {N}}_0$, and it only contributes poles in ${\mathcal {S}}$ in $S_{\frac{1-K+\varepsilon }{3},\infty }$ by Lemma 5.12, where $0<\varepsilon <1$. So the statement about the poles follows if $K\rightarrow \infty$.

We are left to show the polynomial bound. With Lemma 5.13 we obtain the bound for the finite sum, as we chose *K* in terms of $\sigma _0$ and $\sigma _1$. Lemma 5.12 implies the polynomial bound for the integral. $\square$

To apply Theorem 1.4 we require $\zeta _{\mathfrak {so}(5)}(0)$.

### Proposition 5.15

We have $$\zeta _{\mathfrak {so}(5)}(0) = \frac{3}{8}$$.

### Proof

Since $I_M(s; z)$ is holomorphic in *s* for $z\in S_{\mu ,\infty }$ by Lemma 5.8 and $\Gamma (s)$ has a pole in $s=0$,

5.9 $$\begin{aligned} \lim \limits _{s\rightarrow 0} \frac{I_M(s; z)}{\Gamma (s)} = 0. \end{aligned}$$

Let $K\in {\mathbb {N}}$. For $z\in {\mathbb {C}}$ with $\textrm{Re}(z)=K-\frac{1}{2}$ and $m\in {\mathbb {N}}_0$, we have $\pm (z+m)\ne 1$. Hence, $$s\mapsto \left( {\begin{array}{c}-2s-z\\ m\end{array}}\right) \zeta (3s+z+m)\zeta (s-z-m)$$ is holomorphic at $s=0$. This implies that for $z\in {\mathbb {C}}$ with $\textrm{Re}(z)=K-\frac{1}{2}$, we have

$$\begin{aligned} \lim \limits _{s\rightarrow 0} \left( {\begin{array}{c}-2s-z\\ m\end{array}}\right) \frac{\zeta (3s+z+m)\zeta (s-z-m)}{\Gamma (s)} = 0. \end{aligned}$$

Using this, (5.8) with $\varepsilon =\frac{1}{2}$, (5.9), Proposition 2.6 (4), and Lebesgue’s dominated convergence theorem, we obtain, for integers $K\ge 3$,

$$\begin{aligned}{} & {} \lim _{s\rightarrow 0} \frac{6^s}{2\pi i\Gamma (s)}\int _{K-\frac{1}{2}-i\infty }^{K-\frac{1}{2}+i\infty } \Gamma (s+z)\Gamma (-z)\zeta _{{\text {MT}},2}(s,s-z,2s+z) dz \\{} & {} \quad = \frac{i}{72} \int _{K-\frac{1}{2}-i\infty }^{K-\frac{1}{2}+i\infty } \frac{1}{\sin (\pi z)} dz. \end{aligned}$$

Since $$\sin (\pi (z+1)) = -\sin (\pi z)$$ and

$$\begin{aligned} \lim \limits _{L\rightarrow \infty }\int _{K-\frac{1}{2}-i L}^{K + \frac{1}{2}-i L} \frac{1}{\sin (\pi z)} dz = \lim \limits _{L\rightarrow \infty }\int _{K+\frac{1}{2}+i L}^{K - \frac{1}{2}+i L} \frac{1}{\sin (\pi z)} dz = 0, \end{aligned}$$

the Residue Theorem implies that

5.10 $$\begin{aligned}{} & {} \lim _{s\rightarrow 0} \tfrac{6^s}{2\pi i\Gamma (s)}\int _{K-\frac{1}{2}-i\infty }^{K-\frac{1}{2}+i\infty } \Gamma (s+z)\Gamma (-z)\zeta _{{\text {MT}},2}(s,s-z,2s+z) dz \nonumber \\{} & {} \quad = \tfrac{1}{72}{\text {Res}}_{z=K} \tfrac{\pi }{\sin (\pi z)} = \tfrac{(-1)^K}{72}. \end{aligned}$$

In the following we use that $\zeta (s)$ does not have a pole in $s=\pm m$ for $m\in {\mathbb {N}}_{\ge 2}$, implying that $$s\mapsto \left( {\begin{array}{c}-2s-1\\ m-1\end{array}}\right) \zeta (3s+m)\zeta (s-m)$$ is holomorphic at $s=0$. Moreover $$s\mapsto \Gamma (s+k)\left( {\begin{array}{c}-2s-k\\ m\end{array}}\right) \zeta (3s+k+m)\zeta (s-k-m)$$ is holomorphic at $s=0$ for $(k,m)\in ({\mathbb {N}}\times {\mathbb {N}}_0)\backslash \{(1,0)\}$. Thus, using Propositions 2.6 (3) and 2.7 (3) and the fact that $\zeta (-1)=-\frac{1}{12}$ and $\zeta (0) = \frac{1}{2}$, we obtain, with (5.6),

5.11 $$\begin{aligned}&\lim \limits _{s\rightarrow 0} \frac{6^s}{\Gamma (s)} \sum _{k=0}^{K-1} \frac{(-1)^k \Gamma (s+k)}{k!}\zeta _{{\text {MT}},2}(s, s-k, 2s+k)\nonumber \\&\quad = \frac{3}{8}+\frac{(-1)^{K+1}}{72}+\lim \limits _{s\rightarrow 0} I_M(s;0) +\sum \limits _{k=1}^{K-1} \frac{(-1)^k}{k}\lim \limits _{s\rightarrow 0} \frac{I_M(s;k)}{\Gamma (s)}. \end{aligned}$$

Since, by Lemma 5.8, $s\mapsto I_M(s;k)$ is holomorphic at $s=0$ for every $k\in {\mathbb {N}}_0$ and $\frac{1}{\Gamma (s)}$ vanishes in $s=0$, we have

$$\begin{aligned} \lim \limits _{s\rightarrow 0} \frac{I_M(s;k)}{\Gamma (s)} = 0. \end{aligned}$$

Applying the Lebesgue dominated convergence theorem gives $\lim \limits _{s\rightarrow 0}I_M(s;0)=0$, yielding the claim with (5.8), (5.10), and (5.11). $\square$

Furthermore, we need certain residues of $\zeta _{\mathfrak {so}(5)}$.

### Proposition 5.16

The poles of $\zeta _{\mathfrak {so}(5)}$ are precisely $\{\frac{1}{2}\}\cup \{\frac{d}{3}\notin {\mathbb {Z}}:d\le 1\text { odd}\}$. We have

$$\begin{aligned} {\text {Res}}_{s=\frac{1}{2}} \zeta _{\mathfrak {so}(5)}(s) = \frac{\sqrt{3}\Gamma \left( \frac{1}{4}\right) ^2}{8\sqrt{\pi }}. \end{aligned}$$

Moreover for $$d\in {\mathbb {Z}}_{\le 1}\setminus ( - 3{\mathbb {N}}_0)$$,

5.12 $$\begin{aligned} {\text {Res}}_{s=\frac{d}{3}} \zeta _{\mathfrak {so}(5)}(s) = \frac{3^{\frac{d}{3}-\frac{3}{2}}\pi \Gamma \left( \frac{d}{6}\right) \zeta \left( \frac{4d}{3}-1\right) }{2^{\frac{d}{3}-1}(1-d)!\Gamma \left( \frac{d}{3}\right) ^2\Gamma \left( \frac{d}{2}\right) }\left( \frac{d}{3}\right) \left( 1+2^{\frac{2d}{3}-1}\right) . \end{aligned}$$

In particular, we have

$$\begin{aligned} {\text {Res}}_{s=\frac{1}{3}} \zeta _{\mathfrak {so}(5)}(s) = \frac{2^\frac{1}{3}+1}{3^\frac{2}{3}}\zeta \left( \frac{1}{3}\right) . \end{aligned}$$

### Proof

With Lemma 5.12, near $s=\frac{1}{2}$, we can choose $K=1$ in (5.8) and obtain

$$\begin{aligned}{} & {} {\text {Res}}_{s=\frac{1}{2}}\zeta _{\mathfrak {so}(5)}(s)\\{} & {} \quad = \lim _{s \rightarrow \frac{1}{2}} \left( s - \frac{1}{2} \right) \left( 6^s \zeta _{\textrm{MT},2}(s,s,2s) + \frac{6^s}{2\pi i\Gamma (s)} \int _{\frac{1}{2}-i\infty }^{\frac{1}{2} + i\infty } \Gamma (s+z)\Gamma (-z) \zeta _{\textrm{MT},2}(s,s-z,2s+z)dz\right) . \end{aligned}$$

Now, we have

$$\begin{aligned} \lim _{s\rightarrow \frac{1}{2}} \left( s-\frac{1}{2}\right) 6^s\zeta _{{\text {MT}},2}(s,s,2s) = \frac{\sqrt{3}\pi }{2\sqrt{2}}. \end{aligned}$$

On the other hand, we find

5.13 $$\begin{aligned}{} & {} \lim _{s\rightarrow \frac{1}{2}} \left( s-\frac{1}{2}\right) \frac{6^s}{2\pi i\Gamma (s)}\int _{\frac{1}{2}-i\infty }^{\frac{1}{2}+i\infty } \Gamma (s+z)\Gamma (-z)\zeta _{{\text {MT}},2}(s,s-z,2s+z) dz\nonumber \\{} & {} \quad = \lim _{s\rightarrow \frac{1}{2}} \left( s-\frac{1}{2}\right) \frac{6^s\Gamma (3s-1)\zeta (4s-1)}{2\pi i\Gamma (s)}\int _{\frac{1}{2}-i\infty }^{\frac{1}{2}+i\infty } \Gamma (s+z)\Gamma (-z)\Gamma (z+1-s) dz, \nonumber \\ \end{aligned}$$

since $s\mapsto \frac{\Gamma (s+z)\Gamma (-z)\zeta (3s+z)\zeta (s-z)}{\Gamma (s)}$ and $s\mapsto \frac{\Gamma (s+z)\Gamma (-z)I_1(s;z)}{\Gamma (s)}$ are holomorphic if $\textrm{Re}(z)=\frac{1}{2}$. Shifting the path to the left and using [21, 9.113], Proposition 2.6 (1), 15.4.26 of [31], and Proposition 2.6 (4) we obtain that (5.13) equals

$$\begin{aligned} \frac{\sqrt{3}\pi }{2\sqrt{2}}{}_2F_1\left( \frac{1}{2},\frac{1}{2};1;-1\right) - \frac{\sqrt{3}\pi }{2\sqrt{2}} = \frac{\sqrt{3}\Gamma \left( \frac{1}{4}\right) ^2}{8\sqrt{\pi }} - \frac{\sqrt{3}\pi }{2\sqrt{2}}. \end{aligned}$$

This proves the first part of the proposition.

Now, let $d\in {\mathbb {Z}}_{\le 1}\setminus (-3{\mathbb {N}}_0)$ and choose $0<\varepsilon <\frac{1}{3}$, and also $K,M>1-d$. We have, by (5.8),

5.14 $$\begin{aligned} {\text {Res}}_{s=\frac{d}{3}} \zeta _{\mathfrak {so}(5)}(s){} & {} = \lim _{s\rightarrow \frac{d}{3}} \frac{\left( s-\frac{d}{3}\right) 6^s}{\Gamma (s)}\sum _{k=0}^{K-1} \frac{(-1)^k\Gamma (s+k)}{k!}\zeta _{{\text {MT}},2}(s,s-k,2s+k)\nonumber \\{} & {} \quad + \lim _{s\rightarrow \frac{d}{3}} \frac{\left( s-\frac{d}{3}\right) 6^s}{2\pi i\Gamma (s)}\int _{K-\varepsilon -i\infty }^{K-\varepsilon +i\infty } \Gamma (s+z)\Gamma (-z)\zeta _{{\text {MT}},2}(s,s-z,2s+z) dz. \nonumber \\ \end{aligned}$$

Note that $\lim \limits _{s\rightarrow \frac{d}{3}}(s-\frac{d}{3})I_M(s;k)=0$ because of holomorphicity of $I_M$ by Lemma 5.8 and

$$\begin{aligned} \lim _{s\rightarrow \frac{d}{3}} \left( s-\frac{d}{3}\right) \zeta (3s+k+m) = \frac{1}{3}\delta _{m=1-d-k}. \end{aligned}$$

Thus we obtain, by (5.6) and (15.4.26) of [31],

5.15 $$\begin{aligned}&\lim _{s\rightarrow \frac{d}{3}} \frac{\left( s-\frac{d}{3}\right) 6^s}{\Gamma (s)}\sum _{k=0}^{K-1} \frac{(-1)^k\Gamma (k+s)}{k!}\zeta _{{\text {MT}},2}(s,s-k,k+2s) \nonumber \\&\quad = \frac{6^\frac{d}{3}\zeta \left( \frac{4d}{3}-1\right) }{3(1-d)!\Gamma \left( \frac{d}{3}\right) } \left( \sum _{k=0}^{K-1} \frac{(-1)^{k+d+1}\Gamma \left( k+1-\frac{d}{3}\right) \Gamma \left( k+\frac{d}{3}\right) }{k!\Gamma \left( k+\frac{2d}{3}\right) }\right. \nonumber \\&\qquad \left. + \sum _{k=0}^{1-d} (-1)^k\left( {\begin{array}{c}1-d\\ k\end{array}}\right) \frac{\Gamma \left( k+\frac{d}{3}\right) \Gamma \left( 1-\frac{2d}{3} -k\right) }{\Gamma \left( \frac{d}{3}\right) }\right) \nonumber \\&\quad = \frac{6^\frac{d}{3}\zeta \left( \frac{4d}{3}-1\right) }{3(1-d)!\Gamma \left( \frac{d}{3}\right) }\left( \sum _{k=0}^{K-1} \frac{(-1)^{k+d+1}\Gamma \left( k+1-\frac{d}{3}\right) \Gamma \left( k+\frac{d}{3}\right) }{k!\Gamma \left( k+\frac{2d}{3}\right) }\right. \nonumber \\&\qquad \left. + \Gamma \left( 1-\tfrac{2d}{3}\right) {}_2F_1\left( \tfrac{d}{3},d-1;\tfrac{2d}{3};-1\right) \right) \nonumber \\&\quad = \frac{6^\frac{d}{3}\zeta \left( \frac{4d}{3}-1\right) }{3(1-d)!\Gamma \left( \frac{d}{3}\right) }\sum _{k=0}^{K-1} \frac{(-1)^{k+d+1}\Gamma \left( k+1-\frac{d}{3}\right) \Gamma \left( k+\frac{d}{3}\right) }{k!\Gamma \left( k+\frac{2d}{3}\right) }\nonumber \\&\qquad + \frac{3^{\frac{d}{3}-1}\zeta \left( \frac{4d}{3}-1\right) \Gamma \left( 1-\frac{2d}{3}\right) \Gamma \left( \frac{2d}{3}\right) \Gamma \left( \frac{d}{6}\right) }{2^\frac{d}{3}(1-d)!\Gamma \left( \frac{d}{3}\right) ^2\Gamma \left( \frac{d}{2}\right) }. \end{aligned}$$

For the integral in (5.14), we obtain that

5.16 $$\begin{aligned}{} & {} \lim _{s\rightarrow \frac{d}{3}} \frac{\left( s-\frac{d}{3}\right) 6^s}{2\pi i\Gamma (s)}\int _{K-\varepsilon -i\infty }^{K-\varepsilon +i\infty } \Gamma (s+z)\Gamma (-z)\zeta _{{\text {MT}},2}(s,s-z,2s+z) dz\nonumber \\{} & {} \quad = \frac{(-1)^{d+1}6^\frac{d}{3}\zeta \left( \frac{4d}{3}{-}1\right) }{3(1{-}d)!\Gamma \left( \frac{d}{3}\right) }\frac{1}{2\pi i}\int _{K-\varepsilon -i\infty }^{K-\varepsilon +i\infty } \frac{\Gamma \left( z{+}\frac{d}{3}\right) \Gamma \left( z{+}1{-}\frac{d}{3}\right) \Gamma (-z)}{\Gamma \left( z{+}\frac{2d}{3}\right) } dz. \end{aligned}$$

By shifting the path of integration to the left such that all poles of $\Gamma (\frac{d}{3}+z)\Gamma (1-\frac{d}{3}+z)\Gamma (-z)$ except the ones in ${\mathbb {N}}_0$ lie left to the path of integration, we obtain with formula (9.113) of [21]

$$\begin{aligned}&\frac{1}{2\pi i}\int _{K-\varepsilon -i\infty }^{K-\varepsilon +i\infty } \frac{\Gamma \left( z-\frac{d}{3}\right) \Gamma \left( z+1-\frac{d}{3}\right) \Gamma (-z)}{\Gamma \left( z+\frac{2d}{3}\right) } dz\\&\quad = \frac{\Gamma \left( \frac{d}{3}\right) \Gamma \left( 1-\frac{d}{3}\right) }{\Gamma \left( \frac{2d}{3}\right) }{}_2F_1\left( \frac{d}{3},1-\frac{d}{3};\frac{2d}{3};-1\right) + \sum _{k=0}^{K-1} \frac{(-1)^{k+1}\Gamma \left( k+\frac{d}{3}\right) \Gamma \left( k+1-\frac{d}{3}\right) }{k!\Gamma \left( k+\frac{2d}{3}\right) }\\&\quad = \frac{\Gamma \left( 1-\frac{d}{3}\right) \Gamma \left( \frac{d}{6}\right) }{2\Gamma \left( \frac{d}{2}\right) } - \sum _{k=0}^{K-1} \frac{(-1)^k\Gamma \left( k+\frac{d}{3}\right) \Gamma \left( k+1-\frac{d}{3}\right) }{k!\Gamma \left( k+\frac{2d}{3}\right) }, \end{aligned}$$

where the final equality is due to (15.4.26) of [31]. Equation (5.12) follows by this calculation together with (5.14), (5.15), (5.16), and Proposition 2.6 (4). Finally note that (5.12) vanishes for even $d\le 1$. $\square$

Now we are ready to prove Theorem 1.3.

### Proof of Theorem 1.3

Note that by Lemma 5.5 and Theorem 5.14 all conditions of Theorem 1.4 are satisfied (with *L* and $R\notin \frac{1}{3}{\mathbb {N}}$ arbitrary large). As $\zeta _{\mathfrak {so}(5)}$ has, by Proposition 5.16, exactly two positive poles $\alpha :=\frac{1}{2}>\frac{1}{3}=:\beta$, Theorem 4.4 applies with $\ell =3$, and we obtain

$$\begin{aligned} r_{\mathfrak {so}(5)}(n)= & {} \frac{C}{n^b}\exp \left( A_1n^\frac{1}{3}+A_2n^\frac{2}{9}+A_3n^\frac{1}{9}+A_4\right) \\{} & {} \left( 1+\sum _{j=2}^{N+1} \frac{B_j}{n^\frac{j-1}{9}}+O_N\left( n^{-\frac{N+1}{9}}\right) \right) ,\quad (n\rightarrow \infty ). \end{aligned}$$

So we are left to calculate *c*, *b*, $A_1$, $A_2$, $A_3$, and $A_4$. By Proposition 5.15, $\zeta _{\mathfrak {so}(5)}(0)=\frac{3}{8}$ and by Proposition 5.16, ${\text {Res}}_{s=\frac{1}{2}}\zeta _{\mathfrak {so}(5)}(s)$, $\omega _\frac{1}{2}=\frac{\sqrt{3}\Gamma (\frac{1}{4})^2}{8\sqrt{\pi }}$ and $\omega _\frac{1}{3}=\frac{2^\frac{1}{3}+1}{3^\frac{2}{3}}\zeta (\frac{1}{3})$. Hence, by (4.4), we get

$$\begin{aligned} c_1=\frac{\sqrt{3}\Gamma \left( \frac{1}{4}\right) ^2\zeta \left( \frac{3}{2}\right) }{16},\qquad c_2 = 3^{-\frac{5}{3}}\left( 2^\frac{1}{3}+1\right) \Gamma \left( \frac{1}{3}\right) \zeta \left( \frac{1}{3}\right) \zeta \left( \frac{4}{3}\right) . \end{aligned}$$

Moreover, by Lemma 4.3, we have

$$\begin{aligned} K_2 = \frac{2c_2}{3c_1^{\frac{2}{9}}},\quad K_3 = -\frac{c_2^2}{27c_1^{\frac{10}{9}}}. \end{aligned}$$

Now, we compute $A_1$, *C*, and *b* by (1.12) and $A_2$, $A_3$, $A_4$ by Theorem 4.4 and obtain

5.17 $$\begin{aligned} b= & {} \frac{7}{12},\quad C = \frac{e^{\zeta _{\mathfrak {so}(5)}'(0)}\Gamma \left( \frac{1}{4}\right) ^\frac{1}{6} \zeta \left( \frac{3}{2}\right) ^\frac{1}{12}}{2^\frac{1}{3}3^\frac{11}{24}\sqrt{\pi }},\quad A_1 = \frac{3^\frac{4}{3}\Gamma \left( \frac{1}{4}\right) ^\frac{4}{3}\zeta \left( \frac{3}{2}\right) ^\frac{2}{3}}{2^\frac{8}{3}},\qquad \qquad \end{aligned}$$

5.18 $$\begin{aligned} A_2= & {} \frac{2^\frac{8}{9}\left( 2^\frac{1}{3}+1\right) \Gamma \left( \frac{1}{3}\right) \zeta \left( \frac{1}{3}\right) \zeta \left( \frac{4}{3}\right) }{3^\frac{7}{9}\Gamma \left( \frac{1}{4}\right) ^\frac{4}{9} \zeta \left( \frac{3}{2}\right) ^\frac{2}{9}},\quad A_3 = -\frac{2^\frac{40}{9}\left( 2^\frac{1}{3}+1\right) ^2\Gamma \left( \frac{1}{3}\right) ^2 \zeta \left( \frac{1}{3}\right) ^2\zeta \left( \frac{4}{3}\right) ^2}{3^\frac{44}{9} \Gamma \left( \frac{1}{4}\right) ^\frac{20}{9}\zeta \left( \frac{3}{2}\right) ^\frac{10}{9}},\qquad \quad \qquad \qquad \end{aligned}$$

5.19 $$\begin{aligned} A_4= & {} \frac{2^8\left( 2^\frac{1}{3}+1\right) ^3\Gamma \left( \frac{1}{3}\right) ^3\zeta \left( \frac{1}{3}\right) ^3 \zeta \left( \frac{4}{3}\right) ^3}{3^8\Gamma \left( \frac{1}{4}\right) ^4\zeta \left( \frac{3}{2}\right) ^2}.\qquad \qquad \end{aligned}$$

This proves the theorem. $\square$

## Open problems

We are led by our work to the following questions:

1. Is there a simple expression for $\zeta '_{{\mathfrak {so}{(}}5)}(0)$?
2. Can one weaken the hypothesis that $f(n) \ge 0$ for all *n* in Theorem 1.4? An important application would be that the $r_f(n)$ are eventually positive. There are many special cases in the literature (see [12–16]), but to the best of our knowledge no general asymptotic formula has been proved.^9^
3. In [20], Erdős proved by elementary means that if $S\subset {\mathbb {N}}$ has natural density *d* and $\mathbbm {1}_S$ is the indicator function of *S*, then $\log (p_{\mathbbm {1}_{S}}(n))\sim \pi {\sqrt{\frac{2dn}{3}}}$. Referring to Theorem 1.4, can one prove by elementary means that for any $\varepsilon >0$
  $$\begin{aligned} \log \left( r_f(n)\right) =A_1n^{\frac{\alpha }{\alpha +1}}+\sum _{j=2}^MA_jn^{\alpha _j}+O(n^{\varepsilon })? \end{aligned}$$
4. Can one “twist” the products in Theorem 1.4 by $w\in {\mathbb {C}}$ and prove asymptotic formulas for the (complex) coefficients of
  $$\begin{aligned} \prod _{n\ge 1} \frac{1}{\left( 1-wq^n\right) ^{f(n)}} ? \end{aligned}$$
  If $f(n)=n$ or $f(n)=1$, then such asymptotics were shown to determine zero attractors of polynomials (see [3, 4]) and equidistribution of partition statistics see [5, 6]), and the general case of $|w|\ne 1$ was treated by Parry [32]. Nevertheless, all of these results require that $L_f(s)$ has only a single simple pole with positive real part.
5. In Theorem 1.4, can one write down explicit or recursive expressions for the constants $A_j$ in the exponent, say in the case that $L_f(s)$ has three positive poles?
6. Can one prove limit shapes for the partitions generated by (1.8) in the sense of [18, 37]?

## Data Availability

No datasets were generated or analysed during the current study.

## Footnotes

1 Note the well-known typographic error in Wrights asymptotic, he is off by a factor $\sqrt{3}$.

2 Note that these count points in polygons.

3 Note that asymptotics for polynomial partitions were investigated in a more general setting by Dunn–Robles [19].

4 Explicit asymptotic formulas for $p_3(n)$, $p_4(n)$, and $p_5(n)$ are given in Corollary 5.4.

5 Note that Romik used different signs for the constants in the exponential.

6 Throughout we use t he principal branch of the logarithm.

7 We can enlarge the discrete exponent sets at will, since we can always add trivial powers with vanishing coefficients to an expansion. Therefore, from now on we always use this expression, even if the set increases tacitly.

8 Recall that we can consider the sequence $\rho _n$ as a function on ${\mathbb {R}}^+$.

9 The one exception is in Todt’s Ph.D. thesis [36, Theorem 3.2.1]; however, there it is further assumed that $r_f(n)$ is non-decreasing, which precludes the principal application of such an asymptotic.
